# Imaging‐Guided Microscale Photothermal Stereolithography Bioprinting

**DOI:** 10.1002/advs.202500640

**Published:** 2025-03-20

**Authors:** Jingyu Sun, Tianqi Fang, Yuze Zhang, Jue Wang, Huan Han, Tsengming Chou, Junfeng Liang, Dilhan M. Kalyon, Hongjun Wang, Shang Wang

**Affiliations:** ^1^ Department of Biomedical Engineering Stevens Institute of Technology Hoboken NJ 07030 USA; ^2^ Department of Chemical Engineering and Materials Science Stevens Institute of Technology Hoboken NJ 07030 USA; ^3^ Department of Chemistry and Chemical Biology Stevens Institute of Technology Hoboken NJ 07030 USA

**Keywords:** bioprinting, imaging‐guided printing, optical coherence tomography, NIR‐II photothermal initiator, stereolithography

## Abstract

Stereolithography bioprinting relies heavily on costly photoinitiators for polymerization, limiting its potential for further technical advancement to meet growing needs in tissue engineering and regenerative medicine. Thermal initiators, in contrast, are low cost, and rapid growth of the photothermal conversion field offers a wide range of materials and tools to convert light into heat. However, high‐resolution photothermal stereolithography bioprinting remains unattainable due to the difficulty of confining heat in an aqueous environment. Here, this challenge has been fully addressed by establishing imaging‐guided microscale photothermal stereolithography bioprinting (ImPSB). This technique is achieved through building a novel imaging‐guided stereolithography system that provides depth‐resolved visualization of the printing dynamics, creating a unique photothermal initiator in the second near‐infrared window, and developing a new bioink by seeing and controlling the photothermal gelation process. ImPSB achieves a printing resolution of ≈47 µm and generates smooth lines of arbitrarily designed shapes with a cross‐sectional diameter as small as ≈104 µm, representing an unprecedented scale from photothermal aqueous stereolithography. Its cellular biocompatibility in printing both bioscaffold and cell‐laden hydrogel is demonstrated, and its feasibility of transdermal printing is also shown. This work sets a new path for high‐resolution stereolithography bioprinting where the vast photothermal resources can be utilized.

## Graphical abstract

This work establishes imaging‐guided microscale photothermal stereolithography bioprinting, achieving microscale photothermal aqueous printing for the first time. Key innovations include novel imaging‐guided stereolithography, unique NIR‐II photothermal initiator, and new bioink with viscosity modifier. This addresses the long‐standing challenge of confining heat in an aqueous environment during photothermal dynamics, opening the avenue for high‐resolution stereolithography bioprinting with the vast photothermal resources.

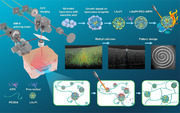

## 1 Introduction

Bioprinting is revolutionizing tissue engineering and regenerative medicine.^[^ ^1^, ^2^ ^]^ A variety of methods, systems, and inks have been developed to address critical limitations in bioprinting,^[^ ^3^, ^4^, ^5^, ^6^ ^]^ advancing significant applications in a range of different biomedical fields, such as wound healing and organ transplantation.^[^ ^7^, ^8^, ^9^, ^10^ ^]^ As one of the major bioprinting techniques, stereolithography bioprinting employs the scanning laser beam for photopolymerization of bioink.^[^ ^11^ ^]^ While this modality has seen rapid growth and success in pushing the boundary of spatial resolution and structural complexity of the print,^[^ ^12^, ^13^, ^14^ ^]^ using photoinitiators remains largely the sole photopolymerization mechanism.^[^ ^15^ ^]^ This restricts stereolithography‐based techniques to rely on only a few available photosensitive molecules,^[^ ^16^ ^]^ yet their high cost and requirement of mainly UV or near‐UV light confine technical innovations and bioprinting strategies.^[^ ^17^ ^]^ Having diverse photopolymerization methods is key to the further development of stereolithography bioprinting to enable novel ideas and approaches.

Photothermal conversion describes an energy transition from light to heat. Its thriving applications in therapy,^[^ ^18^, ^19^, ^20^ ^]^ catalysis,^[^ ^21^, ^22^, ^23^ ^]^ imaging,^[^ ^24^, ^25^, ^26^ ^]^ and motion control^[^ ^27^, ^28^, ^29^ ^]^ have produced extensive materials and tools to achieve efficient light‐to‐heat conversion.^[^ ^30^ ^]^ In addition, a variety of thermal initiators broadly exist, are low cost, and have been commonly used for polymerization.^[^ ^17^ ^]^ While coupling photothermal conversion and thermal initiator presents an appealing path to significantly boosting the photopolymerization method, very few studies have been reported for photothermal stereolithography bioprinting, or even just for photothermal stereolithography printing.^[^ ^31^, ^32^ ^]^

There are two widely acknowledged challenges in this direction: the cells’ inherently low tolerance to heat for biocompatibility^[^ ^33^, ^34^ ^]^ and the difficulty of confining heat for a high‐resolution print.^[^ ^34^, ^35^ ^]^ Regarding the former, Lee et al. showed that a mild photothermal gelation process with near‐infrared light does not have a substantial effect on cell viability, and enhancing the cell–matrix interaction helps to maintain the subsequent cell survival.^[^ ^31^ ^]^ Although this work focused on polymerization instead of printing, it pointed to the potential and possible approaches for photothermal printing of cell‐laden hydrogel. For the latter, two recent studies have started to develop ways of localizing heat that enabled printing in the stereolithography configuration. Lee et al. used a photothermal plate that could be locally heated by a laser beam and could be rapidly cooled down to enable localized resin curing and avoid its outgrowth, respectively.^[^ ^32^ ^]^ Kam et al. employed nanoparticles as a localized light‐to‐heat converter at the near‐infrared range to print complex structures.^[^ ^17^ ^]^ A resolution of 2 µm was achieved with non‐aqueous ink composition,^[^ ^17^ ^]^ which, however, is not suitable for bioprinting. These works pioneered photothermal stereolithography printing, yet the spatial resolution of aqueous printing from both approaches was on the millimeter scale. To the best of our knowledge, microscale photothermal stereolithography bioprinting remained unattainable.

Inspired by these studies,^[^ ^17^, ^31^, ^32^ ^]^ we hypothesized that visualization and monitoring of the localized photothermal dynamics would lead to a better understanding and thus control of the heating and gelation process, which would enable high‐resolution photothermal stereolithography bioprinting. To test this hypothesis, we 1) built a novel imaging‐guided laser‐scanning bioprinting system for simultaneous stereolithography printing and real‐time depth‐resolved imaging, 2) established the major component of bioink to achieve endogenous contrast for label‐free imaging of gelation, and 3) engineered a porous and layered gold‐platinum nanoframework for robust carrying of thermal initiators and efficient photothermal conversion in the second near‐infrared window (NIR‐II). These allowed us to see the process, measure the dynamics, and develop the bioink, and we subsequently established, for the first time to the best of our knowledge, imaging‐guided microscale photothermal stereolithography bioprinting (ImPSB).

**Scheme 1:**
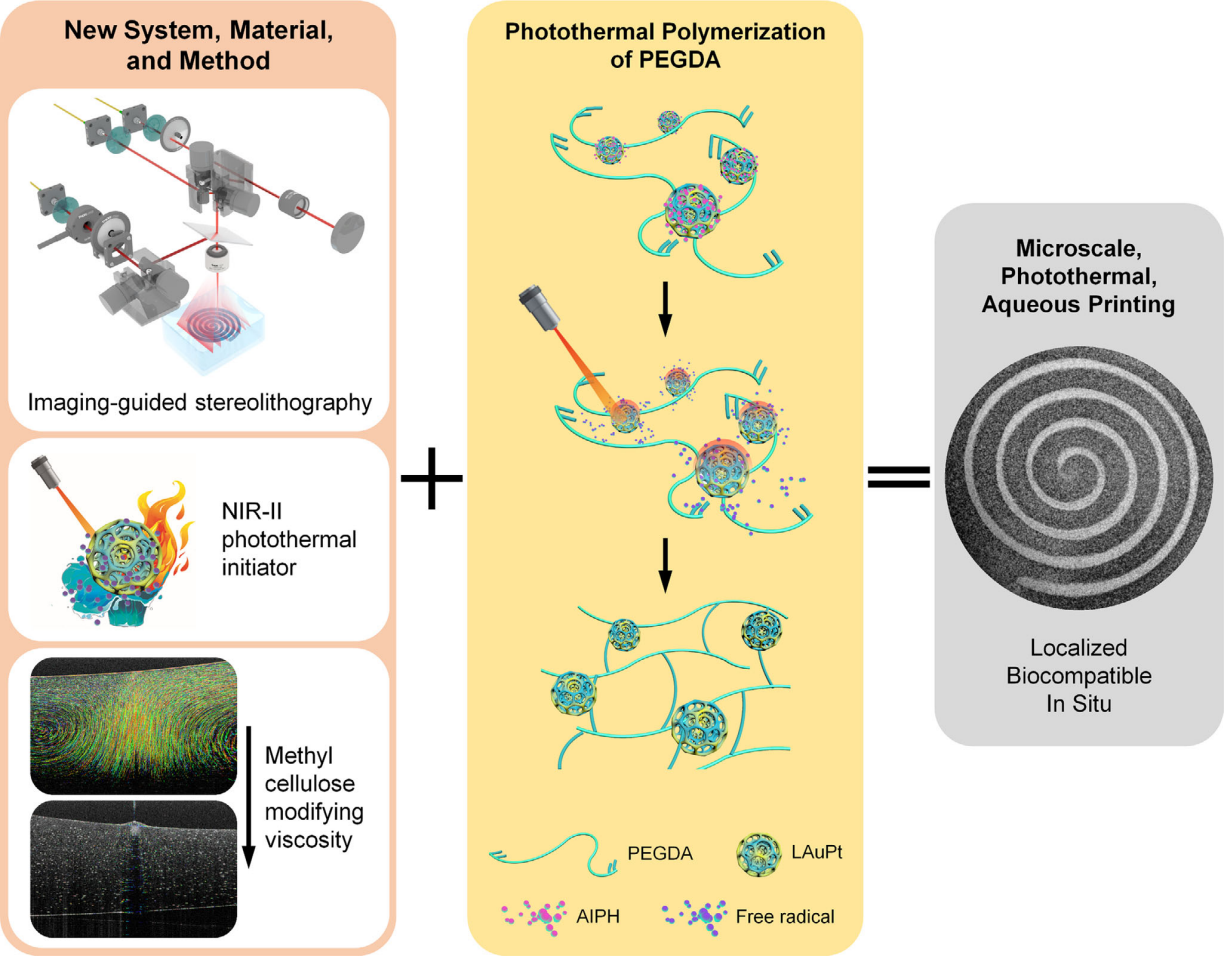
Microscale photothermal stereolithography bioprinting is achieved, for the first time, through building a novel imaging‐guided stereolithography system that integrates optical coherence tomography (OCT) for real‐time, depth‐resolved imaging, creating a photothermal initiator in the second near‐infrared window (NIR‐II), and developing an aqueous bioink with controlled viscosity for efficient, localized photothermal gelation.

**Scheme** **1** illustrates the three major innovations from this work, including the imaging‐guided stereolithography, the NIR‐II photothermal initiator, and the use of methyl cellulose to modify the ink viscosity in response to fluid dynamics, all of which together enabled localized, biocompatible, and in situ photothermal gelation, leading to the microscale photothermal aqueous printing. In terms of imaging, optical coherence tomography (OCT) was employed for ImPSB. OCT relies on low‐coherence light and interferometry to generate a microscale axial resolution, which together with its millimeter‐level imaging depth, fills the gap between high‐frequency ultrasound and confocal microscopy.^[^ ^36^ ^]^ This imaging scale is ideal for visualizing microscale dynamics^[^ ^37^ ^]^ and tissue‐level bioprint.^[^ ^38^ ^]^ In addition, OCT is label‐free with light‐scattering‐based contrast and provides fast 3D imaging based on laser scanning,^[^ ^39^ ^]^ making it particularly advantageous for guiding microscale bioprinting. In terms of light stimulation for photothermal effect, NIR‐II light was selected for ImPSB due to its advantages of increased tissue penetration depth and higher biocompatibility in comparison with the lower‐wavelength light.^[^ ^40^, ^41^ ^]^ Specifically, the relatively longer wavelength of NIR‐II light allows it to have reduced interactions with cells and extracellular matrices in the tissue;^[^ ^42^ ^]^ this leads to its less scattering and absorption from various tissue components, thus making NIR‐II light reach deeper inside the tissue^[^ ^42^ ^]^ and also allowing cells to have a higher tolerance to NIR‐II light (ANSI Z136.1‐2022, American National Standard for Safe Use of Lasers). Therefore, NIR‐II light has significant advantages for bioprinting, especially in situ inside the tissue.

In this paper, we demonstrated ImPSB for its high resolution, arbitrarily designed printing structure, cellular biocompatibility, and transdermal printing capability. This system platform, the ImPSB method, and our studies together open the avenue of applying the vast photothermal resources to revolutionize high‐resolution stereolithography bioprinting.

## 2 Results

### 2.1 ImPSB System

**Figure 1:**
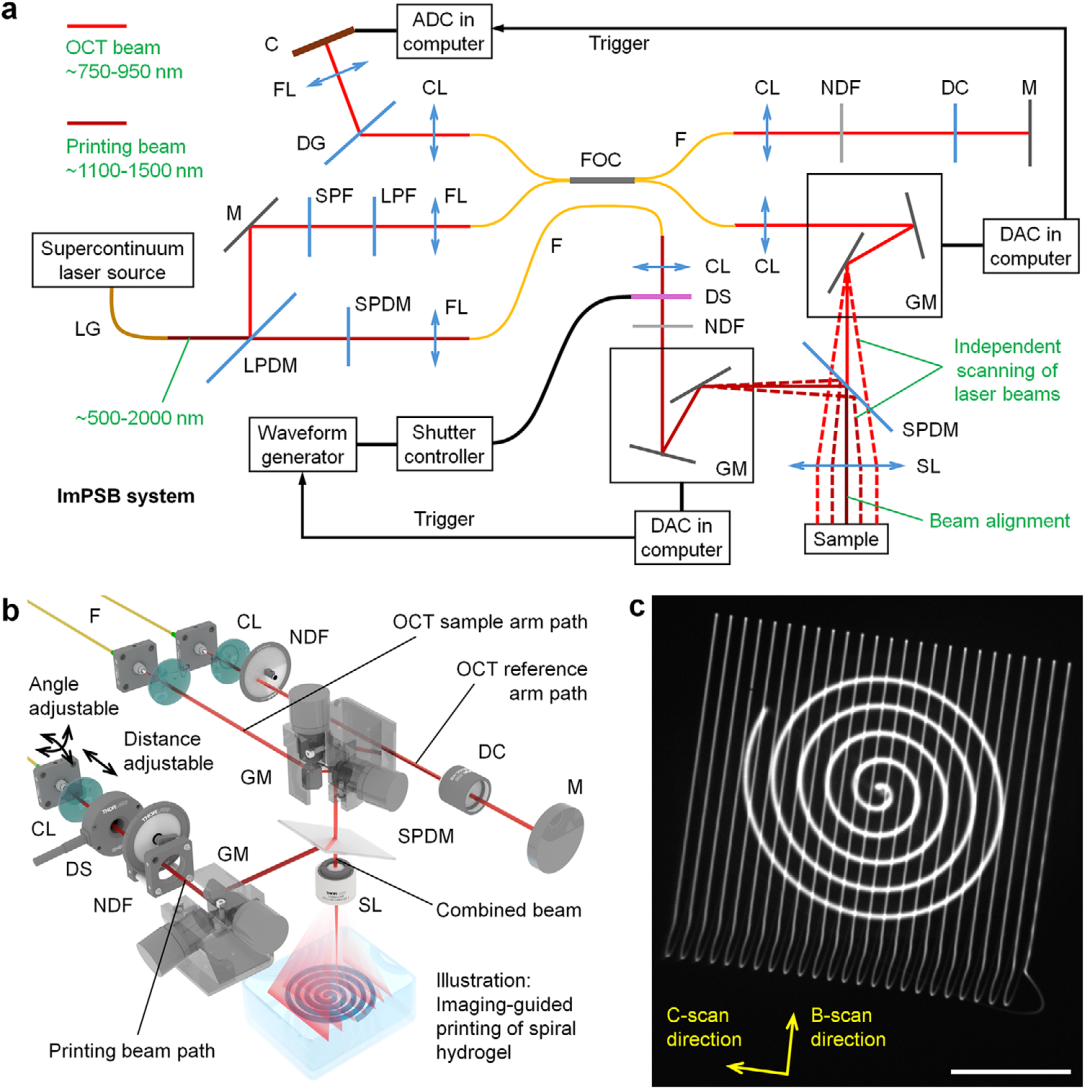
System of ImPSB with independent control of the printing and imaging beams. a) Schematic of the ImPSB system. Dashed lines of optical paths illustrate the beam movement ranges. b) Detailed 3D schematic showing the combination of the two beams with the optical components in their paths. The angle adjustment and the distance adjustment indicated in the printing beam path allow for alignment to overlap the focal spots of the printing and imaging beams. c) A long‐exposure camera image of the two beams providing an example of the independent control of printing and imaging with pre‐alignment of the beams. The number of raster scans was intentionally reduced to better reveal the OCT beam locations. Scale bar is 500 µm. LG: light guide. LPDM: long‐pass dichroic mirror. M: mirror. SPF: short‐pass filter. LPF: long‐pass filter. SPDM: short‐pass dichroic mirror. FL: focusing lens. F: fiber. FOC: fiber optic coupler. CL: collimating lens. NDF: neutral density filter. DC: dispersion compensator. GM: galvanometer mirrors. DS: diaphragm shutter. SL: scan lens. DG: diffraction gratings. C: camera. ADC: analog‐to‐digital converter. DAC: digital‐to‐analog converter.

We designed and built the ImPSB system that combines stereolithography printing capability with OCT. The schematic of the system is shown in **Figure** **1**a. The ImPSB system employs a high‐power supercontinuum laser (SuperK EXTREME EXR‐9 OCT, NKT Photonics) spanning ≈500–2000 nm. Using dichroic mirrors and filters, we separated out the wavelength ranges of ≈750–950 nm for OCT and ≈1100–1500 nm for printing (Figure S1, Supporting Information). The OCT part of the system has a spectral‐domain configuration, featuring a 50/50 fiber coupler for light interference and a high‐speed spectrometer (Cobra‐S 800, Wasatch Photonics) with an A‐scan rate up to 250 kHz. The axial and transverse resolutions of OCT are ≈6 µm in air and ≈9 µm, respectively. Two separate sets of galvanometer‐mirrors (GVS012, Thorlabs) are used for independent lateral scans of the OCT beam and the printing beam; the two laser beams are then combined through a dichroic mirror (DMSP1000R, Thorlabs) and are focused into the sample by the same scan lens (LSM03‐BB, Thorlabs). This maximizes the flexibility for integrated stereolithography printing and OCT imaging, and is suitable for in situ imaging‐guided printing. Notably, the completely separate control of the two laser beams makes the integration of OCT imaging have no effect on the speed of stereolithography printing. Figure 1b illustrates this part of the system in 3D.

The power of the printing beam can be controlled in two ways: a fast on–off switch through a diaphragm shutter (SHB025T, Thorlabs) that controls the start and end of printing, and a gradual power adjustment through a continuously variable neutral density filter wheel with angle labels (NDM4, Thorlabs) that is located only in the printing beam path, allowing the printing light power to be set independently from OCT imaging. The diaphragm shutter is driven by a waveform generator that is triggered by a transistor‐transistor logic signal generated by the digital‐to‐analog convertor (DAC) from the computer, and this same DAC is used to control the set of galvanometer mirrors for the printing beam (Figure 1a). Thus, the galvanometer‐mirrors for printing are synchronized with the shutter to enable precisely timed illumination during the steering of the printing beam. The gradual adjustment of the printing beam power can be made by slowly rotating the continuously variable neutral density filter wheel to introduce a desired amount of attenuation into the printing beam path. In this work, we used a power range of 30–40 mW of the printing beam coming out from the scan lens. The power was measured with a power meter that contains an integrating sphere photodiode power sensor (S145C, Thorlabs) and a digital console (PM100D, Thorlabs), and the wavelength was set as 1300 nm, the center wavelength of the NIR‐II printing laser beam.

A collimated printing beam before the scan lens produces a beam diameter of ≈20 µm at focus. The axial focal location of the printing beam is adjustable by changing the collimation status of beam entering the scan lens, which allows for its flexible axial focusing relative to the OCT imaging beam (Figure S2, Supporting Information). Our test revealed that such an axial focus shift of the printing beam relative to the OCT beam has a range of at least 1.5 mm for both upward and downward directions, which is determined by how much convergence or divergence of the printing beam can be made before the scan lens. We used a camera‐based beam profiler (LBP2‐HR‐VIS2, Newport) to align the two beams after the scan lens. Representative images of the overlapped beams are shown in Figure S3, Supporting Information. With our design of the system, this pre‐alignment provides three important features of imaging‐guided printing. First, overlapping the beams laterally with known settings of galvanometer‐mirrors allows for accurate prediction of the printing beam location through OCT imaging, enabling imaging‐guided printing at particular locations that are of interest inside the sample. Second, by setting the scan area of OCT larger than the entire printing beam path, the printing process can be monitored at a high spatiotemporal resolution with time‐lapse 2D or 3D OCT imaging. These two features can be seen from a long‐exposure image of the two beams shown in Figure 1c, where a spiral path of the printing beam is precisely located and completely covered within a 2D raster scan of the OCT imaging beam. Third, setting a printing beam diameter at the OCT beam focal plane enables the estimation of the printing beam size at the specific depth in the OCT image, allowing for adjustment of the unit printing volume. All these three features are reflected in our results characterizing the printing capabilities of ImPSB.

### 2.2 Endogenous Imaging Contrast for Gelation

For label‐free imaging of the printing process with OCT, the base of the ink needs to provide an OCT imaging contrast before and after gelation. We selected a vinyl oligomer of poly(ethylene glycol) diacrylate (PEGDA, average Mn 700) as the base component, and we used the aqueous solutions of PEGDA with 2,2′‐azobis[2‐(2‐imidazolin‐2‐yl) propane]‐dihydrochloride (AIPH) as the thermal initiator. AIPH decomposes under heat to produce free alkyl radicals that react with the acrylate groups of PEGDA, starting the polymerization process. The propagation continues with the reaction, forming longer chains. The growing chains eventually combine or terminate through reactions such as hydrogen transfer, and this forms a crosslinked polymer network.

**Figure 2:**
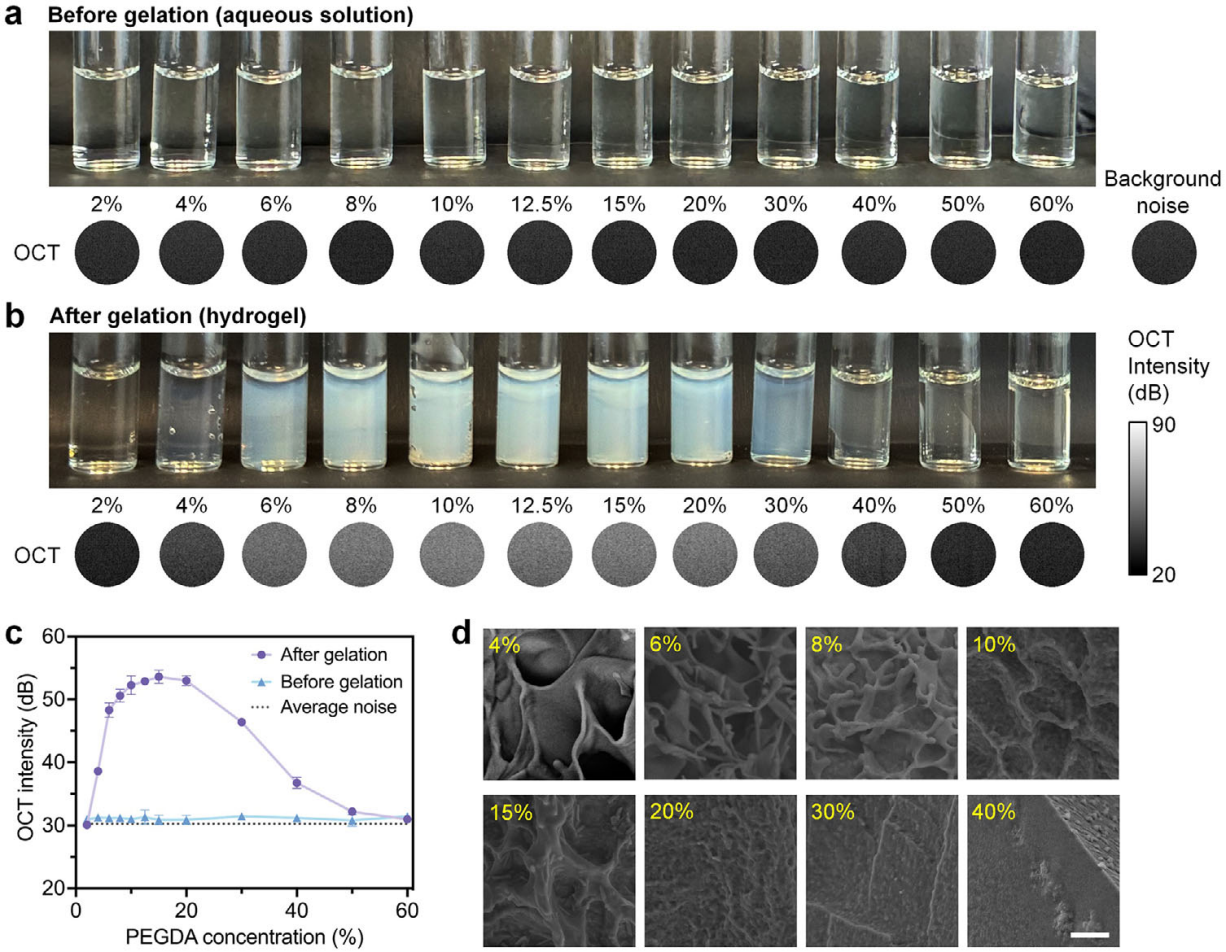
Endogenous contrast for OCT imaging of gelation. a,b) Visual examination and OCT intensity of aqueous PEGDA solutions and PEGDA hydrogels across 2–60% PEGDA concentrations. c) Measurement of OCT intensity before and after PEGDA gelation, showing the OCT imaging contrast over a wide range of concentrations. Data: mean ± std, measurements *N* = 3. d) Scanning electron microscopic (SEM) images revealing the variation of porosity of PEGDA hydrogels from different concentrations. Scale bar is 4 µm.

We show that the OCT imaging contrast can be formed over a wide range of PEGDA concentrations (**Figure** **2**). Specifically, aqueous PEGDA solutions ranging from 2% to 60% are visually transparent (Figure 2a, top), and the OCT intensity signals are at the background noise level (Figure 2a, bottom). The gelation of PEGDA is through an ambient temperature of 44 °C. The light scattering property of the PEGDA hydrogels depends on the PEGDA concentration and reaches a peak at 15%. Specifically, the scattering of the hydrogel increases with the PEGDA concentration increasing from 2% to 15%, and the scattering of the hydrogel decreases with the PEGDA concentration further increasing from 15% to 60% (Figure 2b, top). The scattering of light gives rise to strong OCT intensity signals, which follow the same trend (Figure 2b, bottom). The quantification of the OCT intensity is shown in Figure 2c. In particular, over 4–40% of the PEGDA concentration, the OCT imaging contrast before and after gelation is higher than 5 dB, allowing for easy and direct determination of the ink gelation. Furthermore, this wide range of applicable PEGDA concentration provides the flexibility for designing and tuning the bioink to accommodate specific applications. The light scattering of PEGDA hydrogels is largely due to their porous structure, resulting from the phase separation during polymerization that produces the polymer‐rich phase and the polymer‐poor phase of PEGDA. The porosity appears to be different with different concentrations of PEGDA (Figure 2d), which leads to the observed trend of light scattering and OCT imaging contrast.

### 2.3 NIR‐II Photothermal Initiator

**Figure 3:**
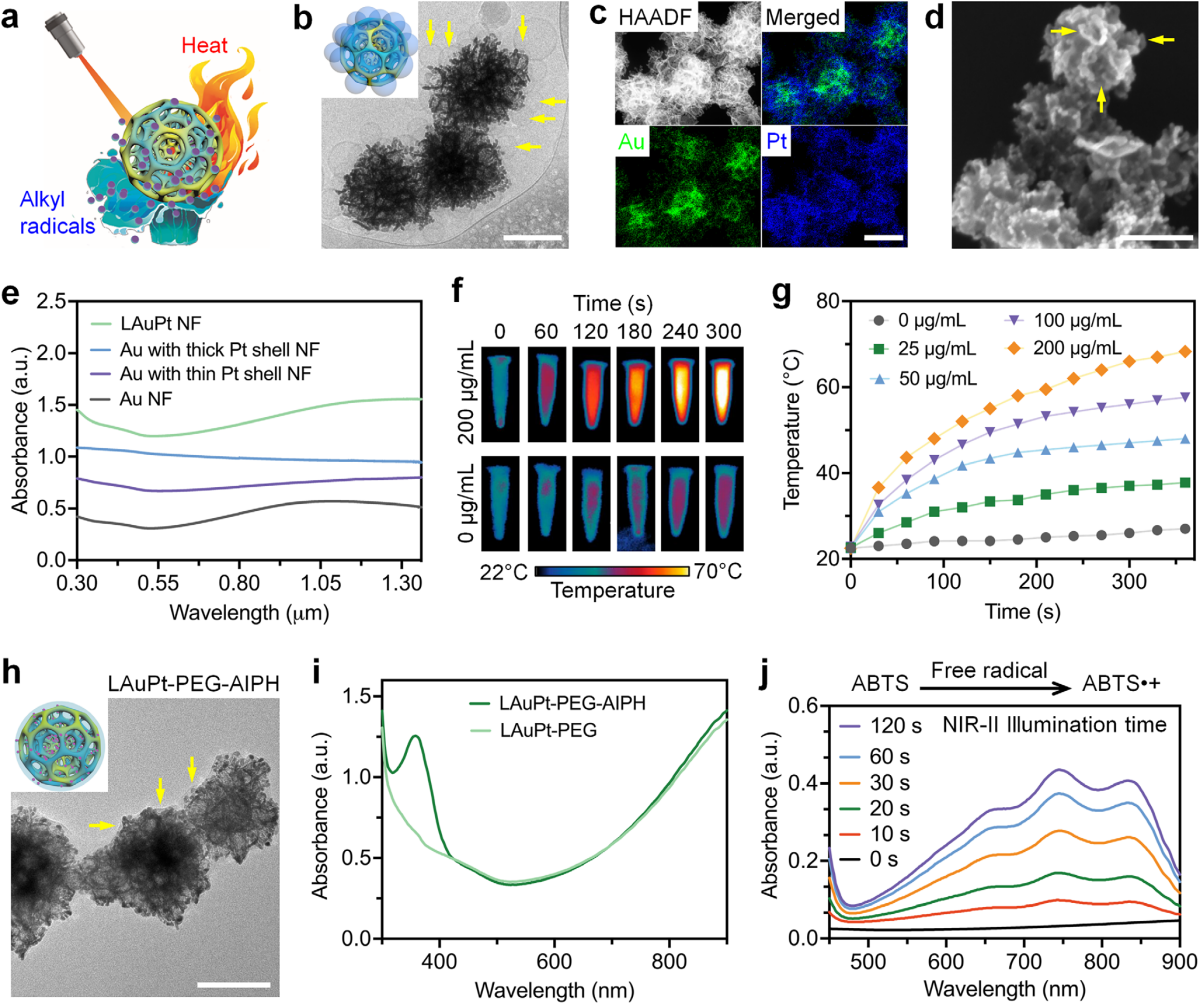
Synthesis and characterization of NIR‐II photothermal initiator. a) Illustration of the NIR‐II photothermal initiator. b) TEM image of the LAuPt nanoframework shows the porous structure and the liposome (arrows) as a template during synthesis. c) High‐angle annular dark‐field (HAADF) scanning TEM image and corresponding energy dispersive X‐ray elemental mapping show the spatial distribution of Au and Pt in the LAuPt nanoframework. d) SEM image of the LAuPt nanoframework shows its porous surface. e) Light absorption spectra of the Au, Au with a thin Pt shell, Au with a thick Pt shell, and LAuPt nanoframeworks (NFs). f) Thermal images of the aqueous solution of LAuPt (200 µg mL^−1^) with ≈1100–1500 nm light illumination over different time durations in comparison with water. g) The changes of temperature from different concentrations of LAuPt aqueous solutions with ≈1100–1500 nm light illumination over different time durations. h) TEM image of the LAuPt‐PEG‐AIPH nanocomposite shows smoother surface (arrows) indicating PEG and AIPH loading. i) Light absorption spectra of LAuPt‐PEG and LAuPt‐PEG‐AIPH confirm the loading of AIPH with the representative peak. j) Light absorption spectra of ABTS solution containing LAuPt‐PEG‐AIPH with ≈1100–1500 nm light illumination over different time periods confirming the release of free alkyl radicals. Scale bars are 100 nm.

For light‐based printing with the thermal initiator AIPH, we engineered a layered gold‐platinum (LAuPt) nanoframework that is porous for carrying AIPH and provides strong photothermal effect in NIR‐II, thus functioning as a NIR‐II photothermal initiator to generate free alkyl radicals upon light exposure (**Figure** **3**a).

The inspiration of creating LAuPt comes from our previous work demonstrating superior NIR absorbance of porous Au nanoframework,^[^ ^43^, ^44^, ^45^ ^]^ as well as other groups’ findings that Pt nanostructures have strong surface plasmonic resonance in NIR‐II.^[^ ^46^, ^47^, ^48^ ^]^ Using the liposome template to regulate the porous structure formation (Figure 3b), we created a three‐layer (Au–Pt–Au) nanoframework (Figure 3c) that has a pore size as large as ≈40 nm (Figure 3d). A 3D visualization is shown in Movie S1, Supporting Information. Measurements through inductively coupled plasma show 56.87% Au and 43.13% Pt in the nanoframework. The LAuPt nanoframework shows further enhanced absorption in NIR‐II, in comparison to the Au nanoframework as well as the AuPt nanoframeworks with different thicknesses of the Pt shell (Figure 3e). Transmission electron microscopy (TEM) images of these four nanoframeworks are shown in Figure S4, Supporting Information, along with their size distribution. Upon exposure to a broad band of ≈1100–1500 nm light, the aqueous solution of LAuPt nanoframework shows a strong photothermal effect with concentration‐ and time‐dependent increases of temperature (Figure 3f,g), and repeated testing of the heating and cooling process indicates its good photostability (Figure S5, Supporting Information). Our further measurement of the photothermal performance shows a high light‐to‐heat conversion efficiency (64.09%) of the LAuPt nanoframework at the concentration of 200 µg mL^−1^.

The porous structure of LAuPt nanoframework provides a high surface‐to‐volume ratio for loading AIPH. We first coated the LAuPt surface with thiol PEG acid via the thiol–Au interaction. Zeta potential measurements (Figure S6, Supporting Information) show that the LAuPt‐PEG nanocomposite has a higher negative surface charge (−26.91 mV) than the bare LAuPt (−6.72 mV), which traps the positively charged AIPH for the loading purpose; after loading, the LAuPt‐PEG‐AIPH nanocomposite shows a near‐neutral surface charge (Figure S6, Supporting Information). Also, adding PEG hinders electrostatic interactions, preventing large aggregation after AIPH loading and showing an improved dispersibility of LAuPt‐PEG‐AIPH in comparison to LAuPt‐AIPH (Figure S7, Supporting Information). The TEM image of LAuPt‐PEG‐AIPH nanocomposites reveals smoother outlines than the bare LAuPt, as well as filled pores (Figure 3h), suggesting the immobilization of PEG onto the LAuPt surface and the infiltration of AIPH into the pores, though without a specific indication of AIPH. We performed additional experiments to specifically detect AIPH and further characterized the successful AIPH loading. First, Fourier‐transform infrared spectroscopy (FTIR) of LAuPt‐PEG‐AIPH indicates the characteristic peak of AIPH at 1597 cm^−1^ (Figure S8, Supporting Information). Second, AIPH has a distinct light absorption peak at 362 nm (Figure S9a, Supporting Information), and the absorption spectrum of LAuPt‐PEG‐AIPH reveals this peak (Figure 3i), in contrast to its absence in the spectrum of LAuPt‐PEG (Figure 3i). Third, we measured the linear relationship between the concentration of AIPH solution and its peak absorption (Figure S9b, Supporting Information), and by assessing the AIPH concentration in the supernatant of the loading solution, we estimated the AIPH loading capacity in LAuPt‐PEG nanocomposite as 67.7% (weight/weight), indicating highly efficient carrying of AIPH.

With the LAuPt‐PEG‐AIPH nanocomposite as a photothermal initiator in NIR‐II, we demonstrated its release of free alkyl radicals upon exposure to ≈1100–1500 nm light. This represents a process of LAuPt converting light into localized heat that decomposes the AIPH it carries to generate free alkyl radicals. We employed 2,2′‐azino‐bis(3‐ethylbenzothiazoline‐6‐sulfonic acid) (ABTS) as a detection probe, which reacts with free alkyl radicals to form the ABTS•+ solution featuring unique absorbance in 500–900 nm.^[^ ^49^ ^]^ Figure 3j shows that LAuPt‐PEG‐AIPH efficiently produces free radicals with ≈1100–1500 nm light illumination; in contrast, illuminating ABTS alone has no effect (Figure S10, Supporting Information).

### 2.4 Dynamic Imaging and Aqueous Printing

**Figure 4:**
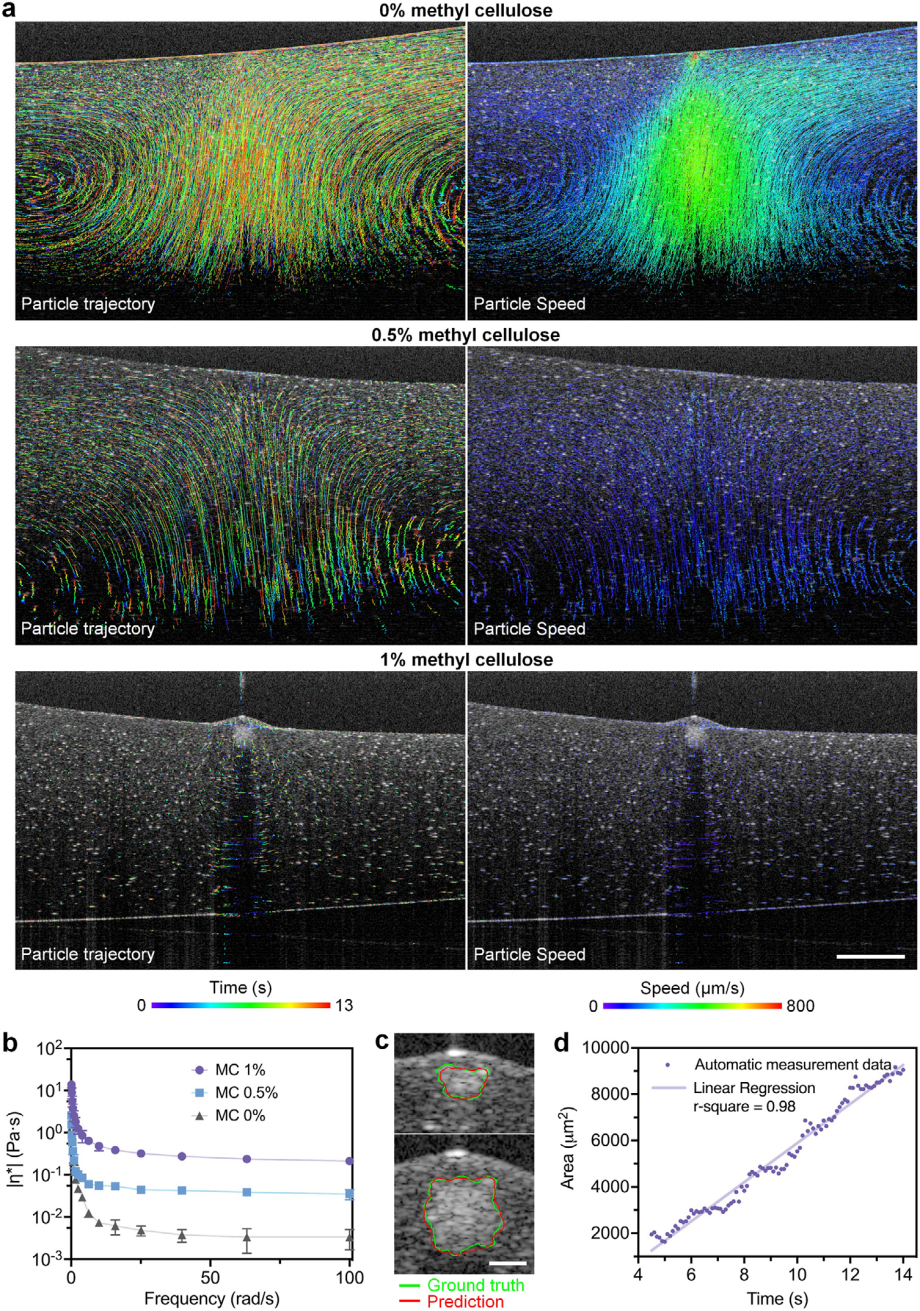
Dynamic imaging and ink development for aqueous photothermal printing. a) Depth‐resolved OCT imaging and measurement of the photothermal dynamics inside the ink, leading to the modification of ink viscosity by methyl cellulose for successful aqueous photothermal printing. b) Dynamic viscosity of the ink with 0%, 0.5%, and 1% methyl cellulose measured through rheology. Data: mean ± std, measurements *N* = 3. c) Two examples show marking the region of hydrogel from a trained deep learning neural network in comparison to the ground truth. d) Automatic quantification of the cross‐sectional area of the hydrogel over time shows the gelation process. Scale bars are 300 µm in (a) and 50 µm in (c).

With the ImPSB system, OCT provides high‐speed, microscale, depth‐resolved imaging inside the ink during point illumination with the printing laser beam. By seeding the ink with 5 µm polystyrene beads, this imaging capability allows us to visualize and understand the photothermal dynamics inside the ink, which has enabled our development of the ink for fast and high‐resolution photothermal printing. In particular, with the aqueous ink only containing LAuPt‐PEG‐AIPH (37 µg mL^−1^) and PEGDA (12.5%), localized illumination with the printing beam induced fluid flows with the peak speed reaching ≈800 µm s^−1^ (**Figure** **4**a; Movie S2, Supporting Information). The heat at the focus of the printing beam led to a specific flow pattern shown by the particle trajectories in Figure 4a. This fluid flow facilitated rapid heat dissipation into the surrounding area away from the printing beam focal spot, preventing localized gelation. Inspired by this observation, we added methyl cellulose to the ink for an increased viscosity to reduce the heat‐induced fluid flow; the measured dynamic viscosity is shown in Figure 4b. With 0.5% methyl cellulose, a weaker flow was observed with a lower speed (Figure 4a), but without localized gelation (Movie S3, Supporting Information). With 1% methyl cellulose, the fluid flow appeared largely absent (Figure 4a), and we achieved rapid gelation at the printing beam focal spot (Movie S4, Supporting Information). The same observation was made with a lower concentration (10% of the original concentration) of the polystyrene beads (Figure S11, Supporting Information), suggesting that seeding the ink does not affect our conclusion. Through this imaging‐guided investigation process, we finalized the basic components of the ImPSB aqueous ink that consists of LAuPt‐PEG‐AIPH (photothermal initiator), PEGDA (base), and methyl cellulose (viscosity modifier). The chemical reaction process is illustrated in Figure S12, Supporting Information.

Having 1% methyl cellulose also improves the stability of the LAuPt‐PEG‐AIPH nanocomposite in the aqueous solution, as shown by a comparison of the nanocomposite precipitation in the aqueous solution with and without 1% methyl cellulose (Figure S13, Supporting Information). During gelation, the peak temperature reaches ≈43.5 °C, which is measured as the spatially averaged temperature over a region of ≈6.6 mm^2^ by a thermal camera (Figure S14, Supporting Information); this spatial scale of measurement is significantly higher than the size of the printing laser beam and the gelation area, and the temperature at the printing beam focal spot is expected to be higher. Furthermore, the PEGDA polymerization is known to generate heat,^[^ ^50^ ^]^ which could in turn contribute to the gelation process; however, for a 0.2 s period of a stationary laser illumination, the heat generated by PEGDA polymerization is estimated to be ≈58.7 µJ, orders of magnitude lower than the heat generated by the photothermal process (on the level of tens of millijoules), suggesting its minimal contribution to the gelation process.

Not only can the OCT imaging guide the ink development but we also show its quantitative monitoring of the localized gelation process with a deep learning neural network. With the OCT imaging contrast (Figure S15, Supporting Information), we trained a U‐Net to successfully mark the region of hydrogel from the ink in the image (Figure S16, Supporting Information). Two examples of the comparison between the computer prediction and the ground truth are shown in Figure 4c. This enables automatic measurement of the gelation size over time (Figure 4d), which indicates an approximately linear growth of the hydrogel cross‐sectional area over the duration of printing beam illumination at the same location.

### 2.5 Resolution and Properties of Printed Hydrogel

**Figure 5:**
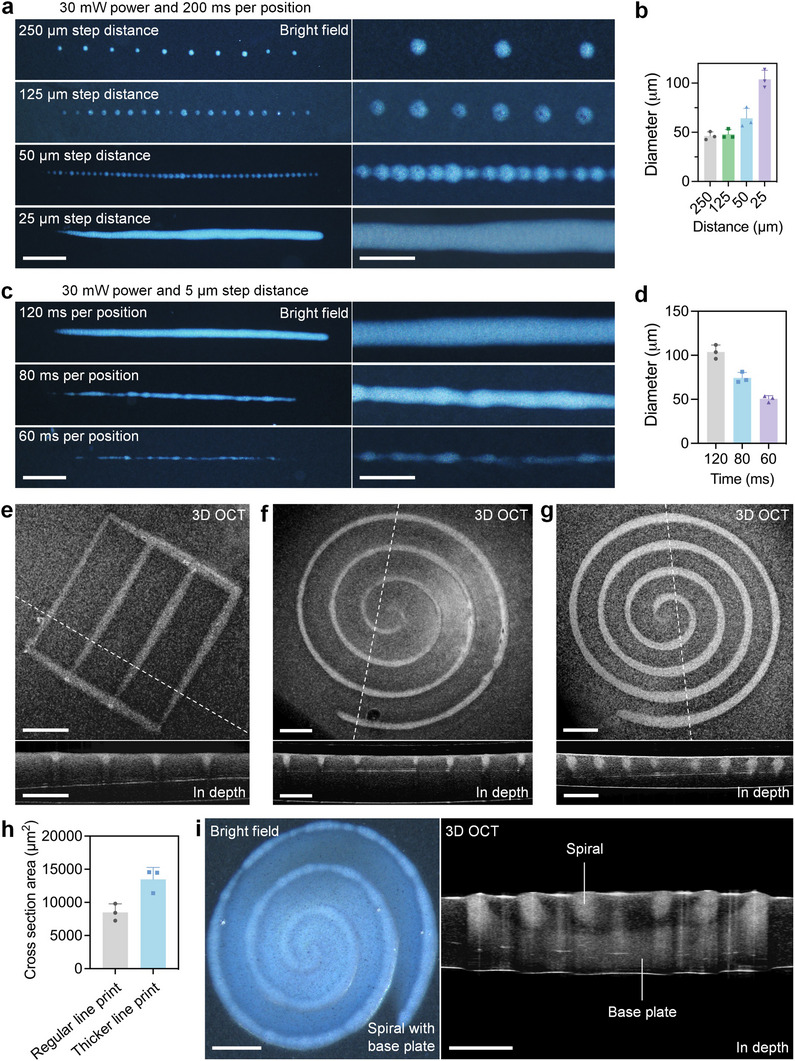
Resolution and structure of a printed hydrogel. a) Bright‐field images of the printed hydrogels of sphere and line with different step distances of the printing beam. b) Measurements of the sphere diameter and line cross‐sectional diameter from different step distances of the printing beam. c) Bright‐field images of the printed line hydrogels with different time durations of illumination at each position of the printing beam path. d) Measurement of the line cross‐sectional diameter from different time durations of illumination at each position of the printing beam path. e) 3D OCT image of a rectangle shape of hydrogel print. f) 3D OCT image of a spiral shape of hydrogel print. g) 3D OCT image of a spiral shape of hydrogel print with an increased line cross‐sectional area. h) Measurement of the line cross‐sectional area from regular and thicker line prints of hydrogel. i) Bright‐field and OCT images of the printed 3D hydrogel structure with spiral line shapes on top of a base plate. Scale bars are 400 µm (left) and 150 µm (right) in (a,c) and 300 µm in (e,f,g,i). Bar plots: mean + std, with data points representing different samples of print.

We define a printed individual sphere as the unit printing volume of ImPSB, which is produced through a point illumination. With an energy density of ≈1.9 kJ cm^−^^2^ for the printing beam, individual spheres of hydrogels with a diameter of ≈47 µm can be repeatably achieved (Figure 5a,b, 250 and 125 µm step distance), which are characterized by 3D OCT images. The 47 µm diameter is considered as the printing resolution of ImPSB, and notably, each of these spheres only takes 0.2 s to print. With the distance between point illuminations at 50 µm, the printed spheres remain separated (**Figure** **5**a, 50 µm step distance), but the spheres appear heterogenous, and the largest sphere reaches a diameter over 47 µm (Figure 5b, 50 µm step distance). This shows that the remaining heat at one printing location affects the printing at adjacent locations when the distance is close to the printing resolution. With the point illumination distance below the resolution (e.g., 25 µm), the moving printing beam produces a smooth line (Figure 5a, 25 µm step distance), and the line cross‐sectional diameter of ≈104 µm can be repeatably achieved (Figure 5b, 25 µm step distance), as characterized by 3D OCT images. We treat the ≈104 µm cross‐sectional diameter as the resolution of ImPSB for line printing. These together demonstrate the microscale printing capability of ImPSB. With the 25 µm point illumination distance, a line of 2.5 mm in length takes 20 s to print, demonstrating a fast printing speed, considering the use of photothermal effect. Our further characterization of the PEGDA cross‐linking reaction through FTIR shows a 61.42% reaction rate, indicating a high printing efficiency of ImPSB.

For line printing, reducing the illumination time at each point while overlapping the printing beam can produce a smooth line of the same characteristics (Figure 5c, 120 ms per position); further reducing the illumination time produces thinner lines, however, with breaking points (Figure 5c,d; 80 and 60 ms per position). This suggests that the energy density, instead of the power density, at each location along the printing path is critical to ensure the continuity and smoothness of the print. With line‐based printing and by programming the transverse path of the printing beam, ImPSB can produce both linear and nonlinear arbitrarily designed shapes, and we show examples of rectangle (Figure 5e) and spiral (Figure 5f), respectively. The 3D OCT imaging of the process of printing a spiral‐shaped hydrogel is shown in Movie S5, Supporting Information. By axially offsetting the printing and OCT beams and placing the printing beam focus deeper into the ink, we achieved a thicker line (Figure 5g) with a larger cross‐sectional area (Figure 5h), demonstrating the adjustable thickness of the line print. Further deepening the printing beam focus into the ink simultaneously prints a base plate and the designed shape, generating a 3D hydrogel structure (Figure 5i). These show the flexibility in adjusting the line‐based printing and highlight the importance of OCT imaging guidance for tuning the printing beam focal position in the ink. Furthermore, the concept of 4D printing^[^ ^51^ ^]^ is employed to explore the capability of ImPSB in generating complex high‐resolution 3D structures. In particular, we find that, when placing the printed line of hydrogel in 1X PBS, the line bends toward the side of laser illumination. This is primarily due to the difference of PEGDA crosslinking density between the two sides (facing and opposite to the laser illumination), which produces unequal swelling of the hydrogel.^[^ ^52^ ^]^ Through this, we successfully generated a high‐resolution 3D spiral structure (Figure S17, Supporting Information).

The hydrogel from ImPSB contains the porous LAuPt nanoframeworks that remain with their original size around 100 nm (Figure S18, Supporting Information), and X‐ray spectroscopy of the hydrogel reveals both Au and Pt components (Figure S19, Supporting Information). These indicate that the LAuPt nanoframework remains intact after printing, allowing for efficient photothermal modulation of the printed hydrogel with NIR‐II light. This photothermal property of the printed hydrogel enables applications such as controlled drug release. As an example, we show that, by adding doxorubicin (DOX) to the ink, the printed hydrogel holds the DOX (Figure S20a, Supporting Information), and illumination with the ≈1100–1500 nm light temporally increases the release rate of DOX from the hydrogel, which is repeatable over time (Figure S20b, Supporting Information).

The mechanical property of the printed hydrogel is tunable based on the concentration of PEGDA in the ink. Both the storage modulus and loss modulus increase with the increase of PEGDA concentration (Figure S21, Supporting Information), covering a wide physiological range. The dominance of storage modulus over loss modulus, coupled with relatively low frequency dependence of both moduli, confirms gel‐like behavior and indicates viscoplasticity, characterized by the presence of a yield stress.^[^ ^53^ ^]^ Yield stress is a crucial property for hydrogels as it signifies the material's ability to resist deformation under low stress conditions.^[^ ^54^ ^]^ This is particularly important in applications requiring structural stability as the printed hydrogel will not deform until the applied stress exceeds this yield threshold. Based on the mechanical property that is associated with the crosslinking density,^[^ ^55^ ^]^ the crosslinking density of the PEGDA through our printing process is measured as 1.31 mol m^−3^.

### 2.6 Cellular Biocompatibility

**Figure 6:**
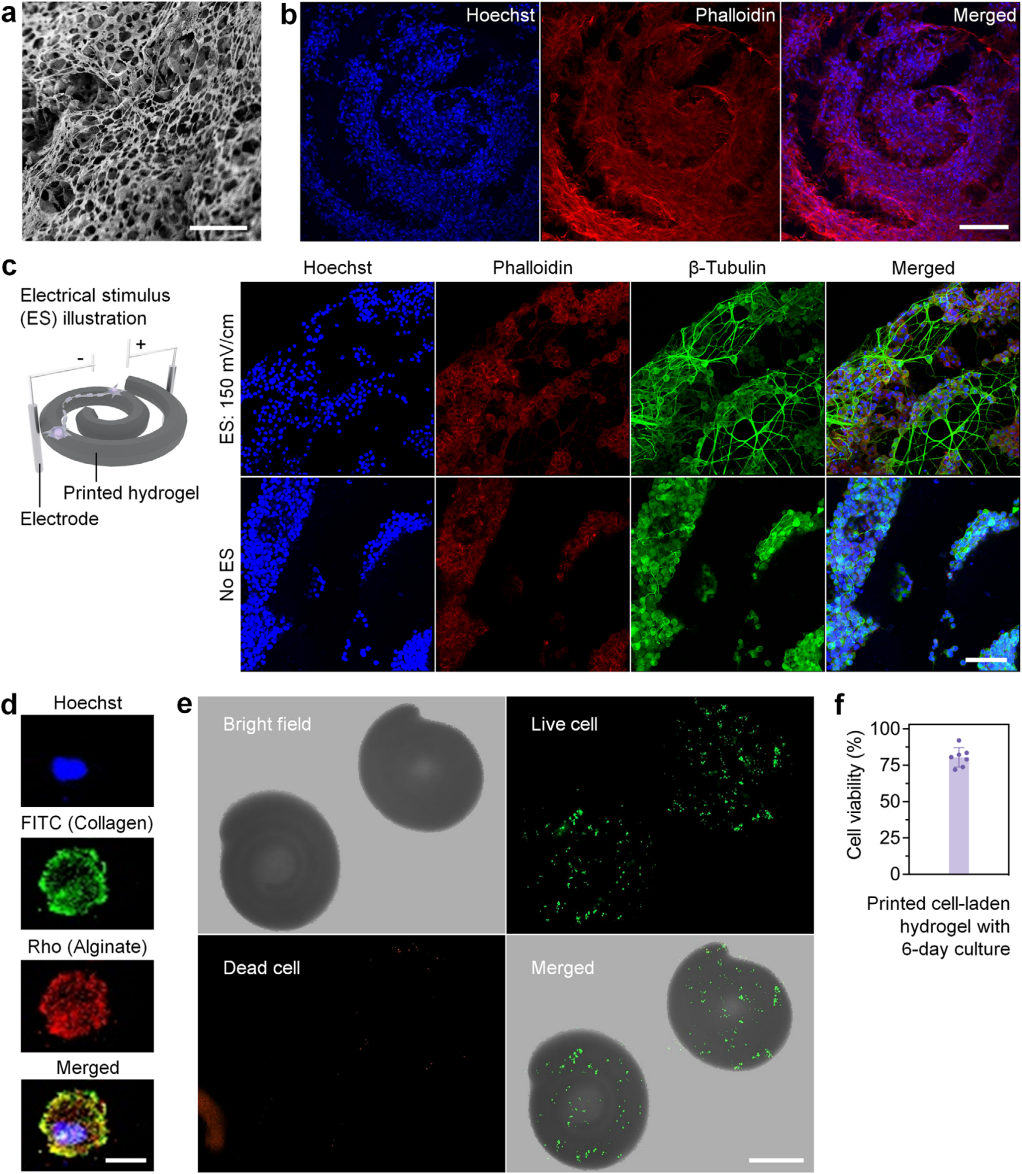
Cellular biocompatibility of printed hydrogel and feasibility of bioprinting. a) SEM image of hydrogel printed from the ImPSB ink containing 10% GelMA. b) Fluorescence images show NIH/3T3 cells form healthy attachment and proliferation on the printed hydrogel as a bioscaffold (7 days after seeding). c) Illustration and fluorescence images show the printed conductive hydrogel as a functional bioscaffold supporting the neurite outgrowth of PC‐12 cells. d) Fluorescence images show the NIH/3T3 cell encapsulation with collagen and alginate, which is used for cell‐laden ink. e) Bright‐field images and live/dead cell staining of hydrogels printed with cell‐laden ink (NIH/3T3 cells, 6 days after print) show the feasibility of bioprinting. f) Quantification of the cell viability from cell‐laden hydrogels (NIH/3T3 cells) shows ≈80% of cells are alive. Scale bars are 20 µm in (a), 600 µm in (b), 100 µm in (c), 15 µm in (d), and 500 µm in (e). Bar plot: mean ± std, with data points presenting different samples of print.

For cellular biocompatibility, we adjusted the ImPSB ink to include 10% gelatin methacrylate (GelMA) that is known to promote cell adhesion, and we kept the PEGDA concentration at 4%. The printed hydrogel shows a highly porous structure, mimicking the extracellular matrix (**Figure** **6**a; Figure S22, Supporting Information), and its mechanical property is shown in Figure S23, Supporting Information. In particular, the porosity of the printed hydrogel (58.4% ± 6.0%) is on the same scale as that of the 0.36 mg mL^−1^ collagen gel (56.7% ± 7.2%), as measured based on SEM images of the 2D cross‐sections of the freeze‐drying samples; the result from this approach is expected to be lower than the porosity measured in 3D. In addition, the Young's modulus of the printed hydrogel (≈1.8 kPa) is comparable with that of the 0.36 mg mL^−1^ collagen gel (≈0.8 kPa), as assessed through micropipette aspiration. With NIH/3T3 cell line, we show that fibroblasts form healthy attachment and proliferation on the printed hydrogel at 7 days post seeding (Figure 6b), demonstrating the feasibility of using the ImPSB print as a bioscaffold. Furthermore, we tested the printed hydrogel as a functional bioscaffold to support specific cell growths. By adding Poly(2,3‐dihydrothieno‐1,4‐dioxin)‐poly(styrenesulfonate) (PEDOT:PSS) to the ink at 1%, we printed bioscaffold with an electrical resistivity of ≈3.5 Ω⋅m, which is considered conductive.^[^ ^56^ ^]^ With electrical stimulation, PC‐12 cells seeded to the scaffold show strong neurite outgrowths forming neuronal‐like network (Figure 6c). This indicates that the ImPSB print can provide functional support to allow cells to react to the proper stimulus.

While the ImPSB ink does not affect cell viability (Figure S24, Supporting Information), when printing with cell‐laden ink, the free alkyl radicals can cause cell death, leading to a low cell viability (Figure S25, Supporting Information). Therefore, we employed the strategy of cell encapsulation^[^ ^57^, ^58^ ^]^ to protect cells from free alkyl radicals as well as heat during the gelation process. In particular, individual NIH/3T3 cells were coated with layers of collagen and alginate (Figure 6d), which were used to prepare the cell‐laden ink. With this approach, ImPSB shows ≈80% cell viability from the printed cell‐laden hydrogels at 6 days after print (Figure 6e), demonstrating the feasibility for bioprinting. The ≈80% cell viability after multiple days of culture is reasonably good in comparison to the major bioprinting techniques with cell‐laden bioinks, which includes the cell viabilities of ≈40–90% for extrusion bioprinting, ≈75–90% for inkjet bioprinting, ≈85–90% for vat‐photopolymerization bioprinting, and ≈90–100% for laser‐induced forward transfer bioprinting, as summarized for the general cases in a recent review.^[^ ^59^ ^]^

### 2.7 Transdermal Printing and Hydrogel Degradation

**Figure 7:**
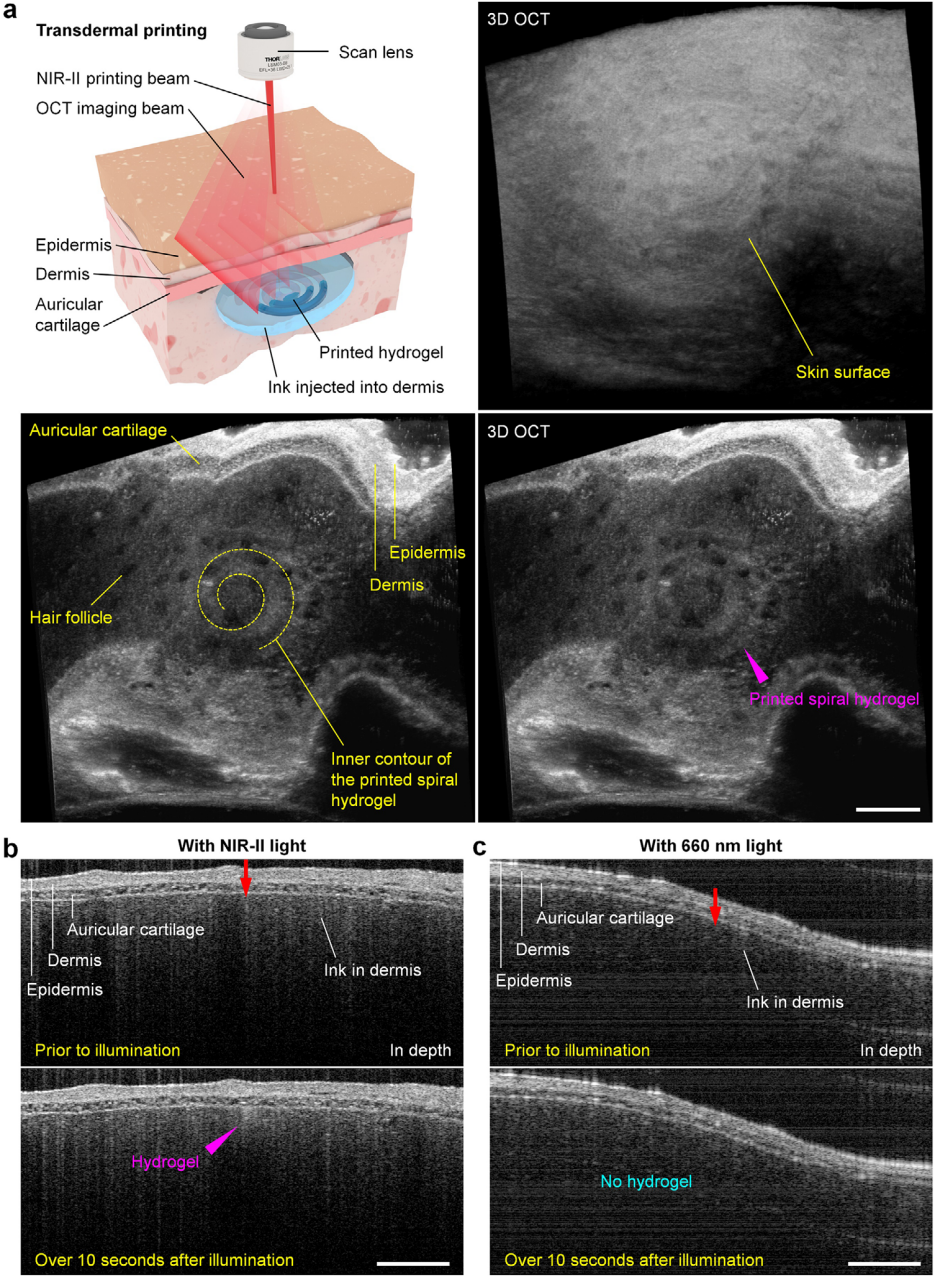
Feasibility of transdermal printing. a) Illustration of transdermal printing and 3D OCT images showing the skin and the printed spiral‐shaped hydrogel under the layers of epidermis, dermis, and auricular cartilage. b,c) A comparison of transdermal printing with light between ≈1100–1500 nm and 660 nm wavelengths shows the higher printing efficiency of using NIR‐II light. Scale bars are 500 µm in (a) and 300 µm in (b,c).

We used the ear skin tissues from euthanized adult mice to show the transdermal printing feasibility of ImPSB (**Figure** **7**). The ink was injected into the dermis layer of the tissue. With the real‐time OCT imaging guidance, the printing beam was focused on the ink under layers of tissue, and a spiral‐shaped hydrogel was printed, which is shown in Figure 7a with 3D OCT images and cross‐sectional views. We further show the advantage of using NIR‐II light with respect to transdermal printing. For comparison, we used gold nanorods (nanoComposix) with a peak absorption at 660 nm (Figure S26, Supporting Information) and 660 nm laser light (OBIS LX, Coherent) to generate localized heating through the photothermal effect. The other components of the ink and the principle of printing remained the same. The concentration of nanorods (55 µg mL^−1^) in the ink was set to have the same photothermal conversion efficiency (at 660 nm illumination) as the LAuPt in the ImPSB ink, and the powers of the respective printing light illumination were set the same. Here, the use of gold nanorods was solely for comparing the transdermal photothermal printing efficiency with using a lower wavelength of light (660 nm). Time‐lapse, depth‐resolved OCT imaging shows that, in comparison to the ImPSB transdermal printing (Figure 7b), no gelation is observed over 10 s of point illumination with 660 nm light (Figure 7c), indicating that the use of NIR‐II light in ImPSB provides a higher printing efficiency through tissue layers.

Based on histology (Figure S27, Supporting Information), the ear skin tissue with hydrogels has intact structure without noticeable defects, especially in the regions (e.g., auricular cartilage) that interface the hydrogel. To evaluate the penetration depth of NIR‐II printing light for printing through optically scattering layers, we used agarose gels containing 5 µm polystyrene beads as the tissue‐mimicking phantoms. With a printing beam power of ≈40 mW out of the scan lens, we performed line printing through tissue‐mimicking phantoms with a range of thicknesses (Figure S28, Supporting Information). Through a thickness of ≈130 µm, the line of hydrogel could be successfully printed, while when the thickness reached ≈390 µm, the printed hydrogel line appeared to have breaking points, indicating unreliable printing. When the thickness reached ≈810 µm, no gelation was observed. These suggest a tissue penetration depth of below ≈810 µm for gelation and a tissue penetration depth of below ≈390 µm for reliable printing with ImPSB employing ≈40 mW of NIR‐II printing light. Using a higher power of the printing beam is expected to increase the penetration depth for reliable printing and gelation.

We also assessed the degradation of the printed hydrogel, as well as the retention of LAuPt nanoframeworks within the printed hydrogel in vitro in the cell culture media. The results indicate no degradation over 15 days, as shown by the volume measurement of the printed hydrogel every 3 days (Figure S29, Supporting Information), and ≈100% retention of the LAuPt nanoframeworks within the printed hydrogel, as shown by the intensity of fluorescence labeling of the LAuPt nanoframeworks (Figure S30, Supporting Information). These suggest the mechanical and photothermal properties of the printed hydrogel can be maintained for at least a period of 15 days.

## 3 Discussion

We have demonstrated ImPSB, which represents the first microscale photothermal stereolithography aqueous printing that is biocompatible for cells; in particular, it allows for printing with cell‐laden ink, indicating the feasibility of bioprinting. The ImPSB technique is enabled by our novel OCT‐guided laser‐scanning printing system, the NIR‐II photothermal initiator, and the ink development through depth‐resolved dynamic imaging guidance. With an interdisciplinary integration and advancement, this work lifts the hurdle that prevents high‐resolution photothermal aqueous printing and opens a new avenue to applying the vastly available photothermal resources for stereolithography bioprinting.

The ImPSB system is the first of its kind by fully integrating OCT with stereolithography, while being able to independently perform time‐lapse 3D OCT imaging and printing beam steering. The pre‐registration of the imaging and printing beams enables accurate positioning of the printing light with OCT depth‐resolved imaging guidance and allows for monitoring, assessing, and understanding of the photothermal gelation process, which were previously not available. As a low‐coherence interferometric imaging modality, OCT provides noninvasive label‐free microscale imaging with a millimeter‐level 3D field of view.^[^ ^36^ ^]^ Although it has been employed for imaging and characterization of the 3D structures of the prints from various approaches,^[^ ^38^, ^60^, ^61^, ^62^, ^63^, ^64^ ^]^ the use of OCT for imaging or studying the printing process was rarely explored. Recently, in a perspective article, Lipkowitz et al. emphasized the value of using OCT for monitoring of the printing process and also presented OCT imaging of the vat photopolymerization process,^[^ ^65^ ^]^ however, without an integrated light delivering system. In contrast, our design and development of the fully integrated OCT‐guided stereolithography system is suitable for in situ printing and paves the way for elucidating the dynamics and processes in a wide range of light‐based printing techniques, not limited to photothermal printing.

The OCT imaging guidance allowed us to develop the ImPSB ink to achieve high‐resolution photothermal printing. Specifically, the observed fluid dynamics upon light illumination indicated that limiting fluid flow is key in confining heat for efficient, localized gelation, which led us to use methyl cellulose to increase the viscosity of the ink. A similar principle was recently utilized by Kuang et al. for the invention of sono‐ink and ultrasound‐based printing.^[^ ^66^ ^]^ Our final composition of the ImPSB ink contains 1% methyl cellulose, enabling fast gelation, and at the same time leaving the ink injectable. Our engineering of the LAuPt nanoframework is another important factor giving rise to the high‐resolution printing. There are several advantages of this novel nanoframework that are closely linked to its functions in ImPSB. First, its size, porous structure, and composition of both Au and Pt generate strong absorbance in the NIR‐II spectrum range for photothermal conversion. Second, the pores of the nanoframework are beneficial for loading AIPH to form a photothermal initiator. Simultaneously providing these two functions makes the LAuPt ideal for ImPSB. The superior NIR‐II photothermal conversion and the large capacity of carrying AIPH create an efficient process from light to heat, to free alkyl radicals, and finally, to the cross‐linking of PEGDA, which is highly localized at each step. Similar chemical reaction processes have been previously used for gelation;^[^ ^31^, ^67^ ^]^ however, printing with the NIR‐II photothermal initiator (LAuPt‐PEG‐AIPH) developed for ImPSB is new. In addition, with the essential ink components including the LAuPt‐PEG nanocomposite, PEGDA, and methyl cellulose, the ImPSB ink can be flexible in its composition; for example, thermal initiators other than AIPH can be employed, and various other matrix materials can be added for different applications.

The print from ImPSB contains the intact LAuPt nanoframeworks, and this photothermal property of the printed hydrogel is important for its applications as a scaffold, such as controlled drug release or where a temperature change plays a significant role in cell manipulations.^[^ ^68^, ^69^ ^]^ In addition, the tunable mechanical property of the print allows for simulating the biomechanical environment provided by a wide range of tissues, which is promising for studying the effect of 3D environmental mechanics on cell activities.^[^ ^70^, ^71^ ^]^ The cellular biocompatibility of the ImPSB hydrogel is largely enabled by an increased cell adhesion, which is enabled by adding GelMA to the ink for the bioscaffold print or through encapsulating cells with collagen and alginate in the cell‐laden ink for bioprinting. In particular, for printing with cell‐laden ink, it is known that free alkyl radicals and heat can cause cell death;^[^ ^72^, ^73^ ^]^ thus, our adoption of the cell encapsulation^[^ ^57^, ^58^ ^]^ also protects cells from such influences, contributing to a high cell viability in the print of cell‐laden hydrogel. For gelation through tissue layers, the use of NIR‐II light presents a higher efficiency than using a lower wavelength of light, and we demonstrated the feasibility of ImPSB for transdermal printing. However, in comparison to transdermal printing with photoinitiators,^[^ ^74^, ^75^ ^]^ photothermal printing is less efficient in terms of the optical energy needed to induce a polymerization. Therefore, even though transdermal photothermal stereolithography printing is possible with NIR‐II light, its clinical usability is limited at the current state, considering the relatively thicker human skin tissue and the generally desired deeper location for printing.^[^ ^76^ ^]^ Yet, this transdermal printing capability of ImPSB could find applications in biological research settings, where in situ bioprinting through layers of tissues is needed. The potential preclinical use of ImPSB for transdermal bioprinting could also benefit from the recent advancements in optical tissue clearing.^[^ ^77^ ^]^

The ImPSB system and method represent a platform technique that can be manipulated and improved in multiple areas for a wide range of applications. Considering the use of photothermal effect, the throughput of ImPSB is already high, featuring five spheres per second for printing individual hydrogel spheres with a diameter of ≈47 µm, as well as 125 µm s^−1^ for printing lines of hydrogels. The printing resolution (the smallest sphere or the thinnest line) is dependent on the bioink composition, and further slowing down the heat dissipation or better confining the heat is expected to lead to an improved resolution. As a higher resolution means less PEGDA is crosslinked to form the hydrogel, less time will be required; and thus, the printing speed or throughput will increase with an improved printing resolution. From the system aspect, our future work will explore the use of space‐division multiplexing^[^ ^78^ ^]^ of the printing beams to improve the printing throughput. On the imaging side, we will incorporate dynamic OCT^[^ ^79^ ^]^ for simultaneous bioprinting and label‐free monitoring of cellular activities. With respect to bioprinting, we will investigate the use of various matrix materials and structures of print^[^ ^80^ ^]^ for studying mechanobiology in 3D. We expect ImPSB to set the foundation for applying photothermal effect to stereolithography bioprinting, diversifying the stereolithography bioprinting strategies and stimulating new ideas in tissue engineering and regenerative medicine.

## 4 Conclusion

We reported the development of ImPSB and demonstrated its unprecedented photothermal stereolithography printing capabilities. By enabling high‐resolution photothermal polymerization in the aqueous environment, ImPSB represents a significant advancement in stereolithography bioprinting. ImPSB features a novel design and implementation of a stereolithography system that fully integrates OCT for real‐time, depth‐resolved imaging of the photothermal printing process, as well as 3D label‐free characterization of the printed hydrogel. With a NIR‐II photothermal initiator, ImPSB achieves a printing resolution of ≈47 µm and produces lines of arbitrarily designed shapes with a cross‐sectional diameter down to ≈104 µm. We presented the flexibility and high efficiency of ImPSB in printing lines with different thicknesses and creating 3D structures, and we showed the unique photothermal property of the printed hydrogel for controlled drug release, as well as its tunable mechanical property covering a wide physiological range. ImPSB has excellent cellular biocompatibility, supporting both bioscaffold and cell‐laden applications. With NIR‐II light, ImPSB has transdermal printing capability and provides the potential for efficient, in situ bioprinting through tissue layers. By leveraging the extensive photothermal resources, ImPSB opens a new pathway for stereolithography bioprinting and broadens the toolkit of high‐resolution bioprinting for tissue engineering and regenerative medicine.

## 5 Experimental Section

### Chemical and Biological Materials

Gold(III) chloride solution, PEGDA, GelMA, PEDOT: PSS, methyl cellulose, cholesterol, L‐ascorbic acid, and Texas red‐conjugated phalloidin were purchased from Sigma–Aldrich (St. Louis, MO, USA). AIPH, DPPC, and Calcein AM/PI staining kit were purchased from Fisher Scientific (Waltham, MA, USA). Doxorubicin was obtained from Cayman Chemicals (Ann Arbor, MI, USA). SH‐PEG2000‐COOH was obtained from Nanocs (New York, NY, USA). All chemicals were used as received without further treatment. Cell lines PC‐12 and NIH/3T3 were obtained from American Type Culture Collection (ATCC, Manassas, VA). Reagents for cell culture, including Dulbecco's phosphate‐buffered saline (DPBS), RPMI 1640 media, Dulbecco's modified Eagle's medium (DMEM), and trypsin/EDTA, were purchased from Gibco (USA). Anti‐beta III tubulin antibody and fluorescein‐conjugated goat anti‐mouse IgG H&L were purchased from Santa Cruz biotechnology (Santa Cruz, CA, USA). The ear skin tissues from euthanized adult mice were used. Mouse euthanasia with carbon dioxide was approved by the Institutional Animal Care and Use Committee at Stevens Institute of Technology (2023‐001).

### Synthesis of LAuPt Nanoframework

Liposome templates were prepared using a modified thin‐film hydration method. In brief, DPPC and cholesterol (molar ratio 55:45) were dissolved in chloroform, and then, the solution was placed under a high vacuum in a rotary evaporator at 55 °C to remove residual organic solvent. The resulting lipid film was hydrated with 10 mL aqueous ascorbic acid solution (0.3 m) at a concentration of 1 mg mL^−1^ via sonication at 55 °C. The sonication could generate cavitation events, which enhanced the dispersion of lipid molecules, and therefore, produced smaller and more uniform liposomes. Unilamellar liposomes were separated by centrifugation at 13 000 rpm for 15 min at 15 °C. Next, 0.2 mL of HAuCl₄ solution (8 mm) was added dropwise to the supernatant containing liposomes. After a 15‐min reaction, 0.2 mL H₂PtCl₆ (10 mm) was introduced, followed by an additional addition of 0.2 mL HAuCl₄ solution (8 mm) after 12 h. The mole ratio of ascorbic acid to metal salt was 3000:5.2. The ascorbic acid as the excessive reactant here denotes electrons during oxidation (converting to dehydroascorbic acid), which reduces gold/platinum ions to gold/platinum nanoparticles (Au⁰/ Pt⁰). The excess reactants and liposome templates were then removed by centrifugation at 10 000 rpm for 10 min, and the final nanostructures were washed three times with deionized water. The obtained mesoporous nanostructures were stored at 4 °C for further use.

### Measurement of Photothermal Conversion Efficiency

The photothermal conversion efficiency of LAuPt was measured with the same approach used by Wang et al.^[^ ^47^ ^]^ Briefly, the aqueous LAuPt solution at 200 µg mL^−1^ was loaded in a quartz cuvette, illuminated with the ≈1100–1500 nm light at 100 mW, and the temperature was measured by a thermometer probe placed inside the solution. The uniformity of the solution temperature was ensured by magnetic stirring. The photothermal effect from water was measured at the same volume (1 mL) and accounted for the assessment of LAuPt. The measurement data from the LAuPt solution are shown in Figure S31, Supporting Information. With the broad band of light illumination, the absorbance at the center (1300 nm) of the wavelength range was used for the calculation.

### Preparing NIR‐II Photothermal Initiator

To prepare the LAuPt‐PEG‐AIPH nanocomposite, the synthesized LAuPt was first functionalized with thiol polyethylene glycol acid (COOH‐PEG‐SH) to enhance biocompatibility and stability in aqueous media. A 20 mg mL^−1^ solution of COOH‐PEG‐SH was prepared, and LAuPt nanoparticles were added to this solution at a 1:10 ratio. The mixture was stirred at room temperature for 12 h to allow COOH‐PEG‐SH to bind to the gold surface via thiol–gold interactions. Following this reaction, the PEGylated LAuPt was centrifuged at 10 000 rpm for 15 min and resuspended in deionized water. This washing step was repeated three times to remove any unbound COOH‐PEG‐SH. Next, AIPH was used as a free alkyl radical generator. A stock solution of AIPH (10 mg mL^−1^) was prepared in PBS. The LAuPt‐PEG was incubated with AIPH at a 1:5 ratio and stirred for 12 h under 4 °C to enable loading. LAuPt‐PEG‐AIPH was then centrifuged at 10 000 rpm for 15 min and washed three times with deionized water to remove any unbound AIPH.

### Measurement of AIPH Loading Capacity

The loading capacity of AIPH in the LAuPt‐PEG nanocomposite was measured indirectly through light spectroscopy. First, AIPH solutions at concentrations of 3.75, 7.5, 18.75, 37.5, 75, 112.5, and 150 µg mL^−1^ were prepared and their absorption spectra over the UV–visible range were obtained. The peak absorption was plotted over the concentration for their linear relationship, through which, a peak absorption value could be used to predict the concentration of AIPH. Then, after the LAuPt‐PEG nanocomposites were mixed with an AIPH solution for AIPH loading, the supernatant post centrifugation was measured by the spectrometer, and the AIPH amount in the supernatant was determined through the linear relationship. The loading capacity of AIPH in LAuPt‐PEG was calculated as the ratio of (original mass of AIPH − mass of AIPH in supernatant) to the total mass of LAuPt‐PEG.

### Measurement of PEGDA Polymerization Efficiency

Fourier transform infrared spectroscopy (FTIR) was used to characterize the PEGDA cross‐linking efficiency. Both ImPSB ink and printed hydrogel were freeze‐dried and assessed with FTIR, and their spectra are shown in Figure S32, Supporting Information. The reduction of the acrylate double bond peak at 810 cm^−1^ was used to represent the polymerization, and its change was quantified to measure the polymerization. The C═O peak at 1721 cm^−1^ was selected as the reference peak to normalize the changes at 810 cm^−1^ for comparison because the C═O was unaffected by the polymerization process. The polymerization efficiency was calculated as the ratio of [(*A*_810_ */A*_1721_)_ink_ − (*A*_810_/*A*_1721_)_hydrogel_] to (*A*_810_ */A*_1721_)_ink_, where *A* is the value obtained from FTIR spectra.

### Cell Encapsulation

Encapsulation of NIH/3T3 cells with collagen and alginate was performed through a modified method of the previous ones.^[^ ^57^, ^81^ ^]^ For the collagen layer, 120 µL of 5× DMEM solution was mixed with 480 µL of 0.03% collagen solution. This mixture was used to resuspend 1 × 10⁶ NIH/3T3 cells, and the suspension was gently shaken for 15 min on ice, allowing the collagen to coat the cells by interacting with the α2β1 integrin receptors on the cell surface. The cells were then centrifuged to remove any excess collagen. After centrifugation, the cells were collected and incubated at 37 °C for 15 min, and the cells were subsequently resuspended in culture medium. For the alginate layer, a similar procedure was followed. However, 1 mL of 0.1% alginate solution was added to the cell pellet, and the tube was gently shaken for 15 min to enable the coating layer to form via electrostatic interactions. After incubation, the cells were centrifuged, and the supernatant was discarded. To visualize the cell coating layer, the coating materials were replaced with FITC labeled collagen and Rhodamine labeled alginate.

### Rheology of Hydrogel and Ink

The linear viscoelastic properties of the hydrogel and ink were characterized using small‐amplitude oscillatory shear experiments on an advanced rheometric expansion system (ARES) rheometer (TA Instruments). A parallel‐disk geometry was employed, utilizing 50 mm disks for the ink samples and 8 mm disks for the hydrogel samples. During oscillatory shearing, a sinusoidal strain, *γ*, was applied over time, *t*, at the frequency, *ω*, that is, *γ* (*t*) = *γ*_0_ sin(*ω* *t*), where *γ*_0_ is the strain amplitude. The measured shear stress, *τ*(*t*), responded to this oscillatory strain with contributions from the storage modulus, *G*′(*ω*), and the loss modulus, *G*′′(ω), expressed as *τ* (*t*) = *G*′ (*ω*)*γ*_0_sin (*ω* *t*) + *G*′′(*ω*)*γ*_0_cos(*ω* *t*). In the linear viscoelastic regime, these moduli are independent of the strain amplitude, with *G*′ and *G*′′ reflecting the elastic energy stored and the energy dissipated as heat, respectively, during each shear cycle. For the ink samples, the magnitude of the complex viscosity, |*η* ^*^|, was calculated as $|\eta^{*}|=\sqrt{{\left(G^{\prime }/\omega \right)}^{2}+{\left(G^{\prime \prime }/\omega \right)}^{2}}$.

### Micropipette Aspiration

The micropipette aspiration was performed with a customized setup that contained a pressure control system (Fluigent), a manual manipulator (World Precision Instruments), an inverted bright field microscope (Primovert, Zeiss) with a color camera (Axiocam 208, Zeiss), and a micropipette (BioMedical Instruments). The micropipette had an inner diameter of 40 µm. 1X PBS was used to fill the micropipette, and the measurement was conducted at room temperature. During aspiration, stepwise increasing pressures, *P*, were applied (up to 2.4 kPa), and the image of the aspiration was taken with a 20× objective. The length of the sample aspirated into the micropipette, *L*, was measured based on the image. A previously established linear relationship was employed between *P* and *L* to quantify the Young's modulus, *E*, of the sample:

(1) $$P=\frac{2\pi E}{3\varphi R}\times L$$

where φ  =  2 as a shape factor and *R* is the radius of the micropipette inner lumen.^[^ ^82^ ^]^ Specifically, a linear regression of *P* versus *L* was performed, and the slope value for the calculation of *E* was obtained.

### Porosity Measurement Based on SEM Images

The porosity of the printed hydrogel and the collagen gel was measured in 2D based on the SEM images of the cross‐sections from the freeze‐drying samples. First, the regions of interest (ROI) with a size of ≈3.7 µm × ≈3.7 µm were selected from the SEM images, covering the porous areas of the sample; then, the images within the ROI were processed through a median filter when noise reduction was needed; next, the images were binarized to highlight the fibers; finally, the total number of the pixels in the black region divided by the total number of pixels in the image was calculated as the porosity. The measurement was performed with ImageJ and MATLAB. This method represents a 2D method for porosity measurement, and the result is expected to be lower than the porosity measured in 3D.

### Measurement of Electrical Resistivity

The hydrogel was cut into cubes with a length of 0.5 cm and was placed in 1X PBS for measurement. The two probes of a digital multimeter were attached to the two sides of the hydrogel cube, and the resistance value, *R*, was obtained. The resistivity of the hydrogel, *ρ*, was calculated as *ρ*  =  *R* × *A*/*L*, where *A* is the cross‐sectional area (0.25 cm^2^), and *L* is the length (0.5 cm).

### Laser Beam Alignment

A camera‐based beam profiler (LBP2‐HR‐VIS2, Newport) was used to align the printing beam to overlap with the OCT imaging beam at the focal spot. The beam profiler provides a 2D view of the laser beam profile, as well as its location in the camera field of view (7.1 mm × 5.3 mm). It is designed for 190–1100 nm but is also sensitive to NIR‐II light, though with a reduced sensitivity. The low sensitivity of NIR‐II light does not affect its use for beam alignment. The two beams were first set with the original, known positions of the galvanometer mirrors (e.g., 0 V for all the mirror motors), and the printing beam was then blocked, with the OCT beam as the only output beam from the scan lens. The distance of the beam profiler relative to the scan lens was then adjusted to have the smallest size of the beam shown from the profiler, indicating the focal spot. This distance was kept during alignment of the printing beam. After this, the printing beam was unblocked, and two types of adjustment were made to the printing beam for alignment (Figure 1b). First, the collimation status of the printing beam entering the scan lens was adjusted to have the smallest beam size shown from the profiler, indicating the focal spot. Second, the angle of the printing beam entering the scan lens was adjusted to overlap with the OCT beam, shown from the camera field of view (Figure S3, Supporting Information). The precision of the alignment is determined by the pixel resolution of the camera, which is 3.69 µm. Given the size of the printing beam at focus (≈20 µm), this pixel resolution provides a relatively high alignment precision.

### Bright‐Field and Fluorescence Imaging

Bright‐field imaging with ZEISS Stemi 508 was performed to observe the printed hydrogel. Fluorescence imaging was performed either by a BioTek Cytation C10 Confocal Imaging Reader or a ZEISS 880 Confocal Laser Scanning Microscope.

### OCT Imaging and Particle Tracking

Both 2D (B‐scan) and 3D OCT imaging were performed in this work. For the OCT intensity A‐scan, the standard linear‐*k*‐space resampling of the interference fridges was performed prior to the fast Fourier transform, and the intensity OCT signal was used for structural imaging. The Imaris software (Oxford Instruments) was used for visualization of the OCT images and tracking of the particles. Specifically, clipping planes were used to visualize particular structures, and the object detection and tracking motion function was utilized to generate particle trajectories over time and map the speed onto the trajectories.

### Deep‐Learning‐Assisted Measurement of Gelation

A time series of B‐scan OCT images of the gelation process was used for training, validating, and testing of deep‐learning neural networks for automatic segmentation of the printed hydrogel. A total of ten images at different time points (showing different sizes of the hydrogel) were selected as a training set. The images were shuffled and divided into two sets, allocating 90% for training and reserving 10% for validation. Data augmentations, including randomly cropping, random 90° rotation, flipping, and random adjustments of brightness and contrast, were used on the training set. A total of 86 images at different time points, excluding the images from the training set, were used to test the segmentation performance. Four architectures from the U‐Net family, including U‐Net, UNet++, nnU‐Net, and Attention U‐Net, were implemented for representativeness and diversity. The input image underwent segmentation without requiring a manual selection of the regions of interest, ensuring a fully automated process. All experiments were implemented with PyTorch 2.2.2 on a NVIDIA GeForce RTX 3060 GPU with details shown in Table S1, Supporting Information. The segmentation results were compared with the manual ground truth and evaluated using Dice Coefficient, Intersection over Union, Accuracy, Specificity, Sensitivity, and Precision, as summarized in Table S2, Supporting Information.

### Statistical Analysis

This study focused on presenting the development of ImPSB and demonstrating its unique capabilities in microscale photothermal bioprinting. No statistical test was involved in this study. Where applicable, the data are shown with the mean and the standard deviation.

## Conflict of Interest

The authors declare no conflict of interest.

## Author Contributions

J.S. and S.W. conceived the ImPSB method and designed the study. J.S., T.F., and S.W. built the ImPSB system. J.S. synthesized the nanoframework, created the NIR‐II photothermal initiator, developed the ImPSB ink, conducted the experiments, analyzed the data, and performed the investigations. T.F., Y.Z., J.W., H.H., T.C., J.L., D.M.K., H.W., and S.W. contributed to the experiments, data analyses, and investigations. J.L., D.M.K., H.W., and S.W. provided resources. S.W. supervised the study. J.S. and S.W. wrote the manuscript with contributions from all authors.

## Supporting information



Supporting Information

Supplemental Movie 1

Supplemental Movie 2

Supplemental Movie 3

Supplemental Movie 4

Supplemental Movie 5

## Data Availability

All data are available within the article and Supporting Information, or available from the corresponding author upon reasonable request.
