# Supplementary material for: Imaging‐Guided Microscale Photothermal Stereolithography Bioprinting
Source: Adv Sci (Weinh). 2025 Mar 20;12(18):2500640. doi: 10.1002/advs.202500640 (PMC12079345; doi:10.1002/advs.202500640)
Supplement: Supplementary file 1 — Supporting Information [file ADVS-12-2500640-s003.pdf]

ADVANCED SCIENCE
Open Access

Supporting Information

for *Adv. Sci.*, DOI 10.1002/advs.202500640

Imaging-Guided Microscale Photothermal Stereolithography Bioprinting

*Jingyu Sun, Tianqi Fang, Yuze Zhang, Jue Wang, Huan Han, Tsengming Chou, Junfeng Liang, Dilhan M. Kalyon, Hongjun Wang and Shang Wang**

Supporting Information

Imaging-guided microscale photothermal stereolithography bioprinting

Jingyu Sun[1], Tianqi Fang[1], Yuze Zhang[2], Jue Wang[3], Huan Han[1], Tsengming Chou[2], Junfeng Liang[3], Dilhan M. Kalyon[2], Hongjun Wang[1], and Shang Wang[1,*]

[1]Department of Biomedical Engineering, Stevens Institute of Technology, Hoboken, New Jersey, 07030, USA
[2]Department of Chemical Engineering and Materials Science, Stevens Institute of Technology, Hoboken, NJ 07030, USA
[3]Department of Chemistry and Chemical Biology, Stevens Institute of Technology, Hoboken, New Jersey, 07030, USA

*Correspondence: shang.wang@stevens.edu

The supporting information includes:

Figures S1-S32

Tables S1-S2

Description of Movies S1-S5

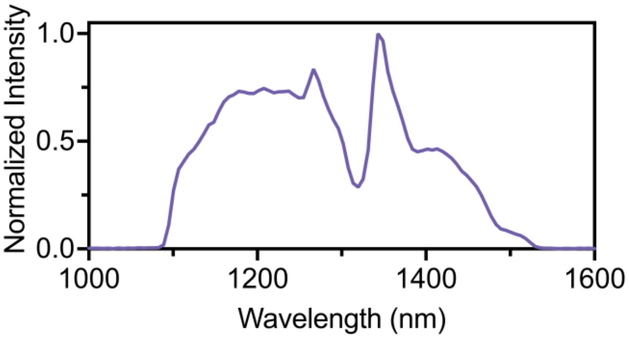

Figure S1: Normalized spectrum of the NIR-II printing light used for ImPSB, featuring the wavelength range of ~1100-1500 nm.

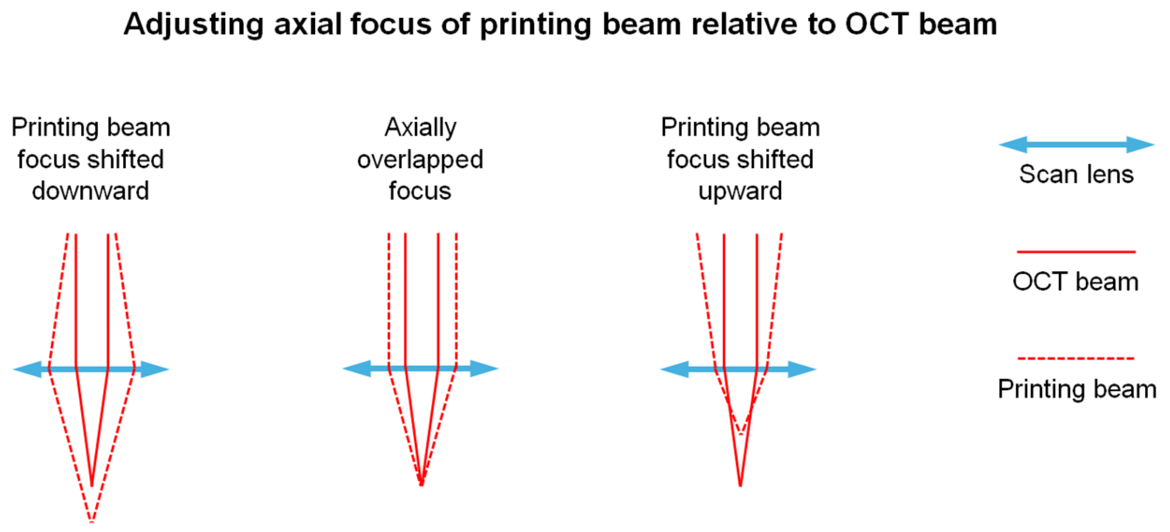

Figure S2: An illustration of shifting the printing beam focus in the axial direction relative to the OCT beam focus through changing the printing beam collimation status before the scan lens.

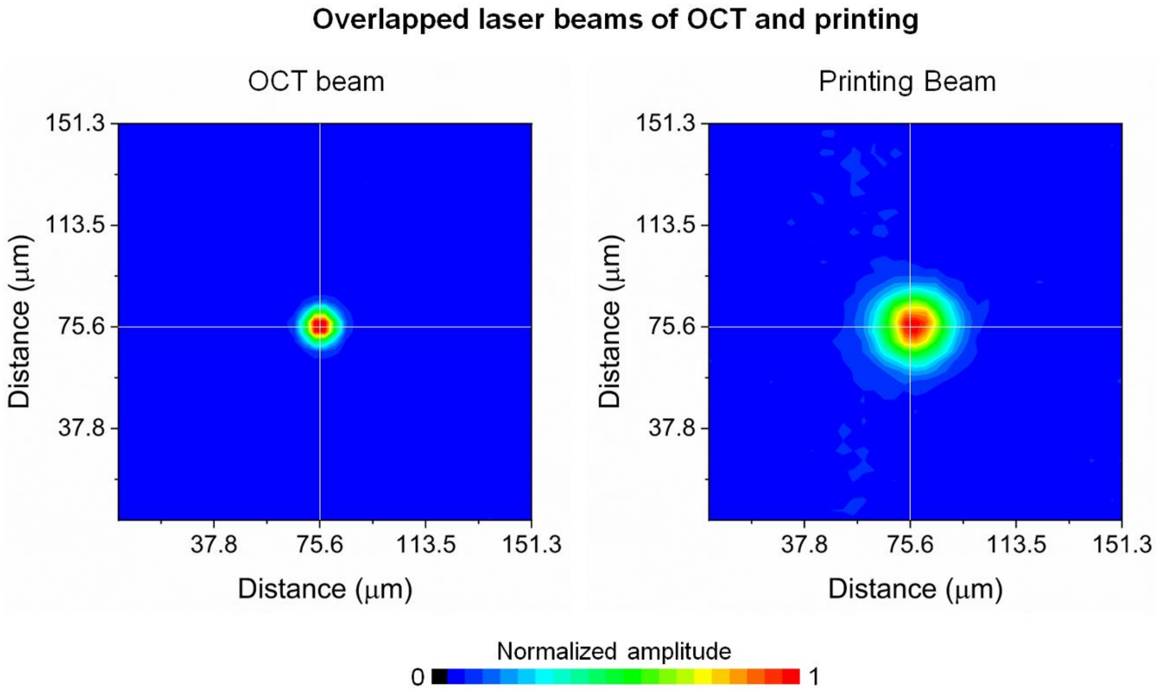

Figure S3: Separated images from the camera-based beam profiler showing the overlapped OCT and printing beams. The pixel resolution of alignment is 3.69 μm.

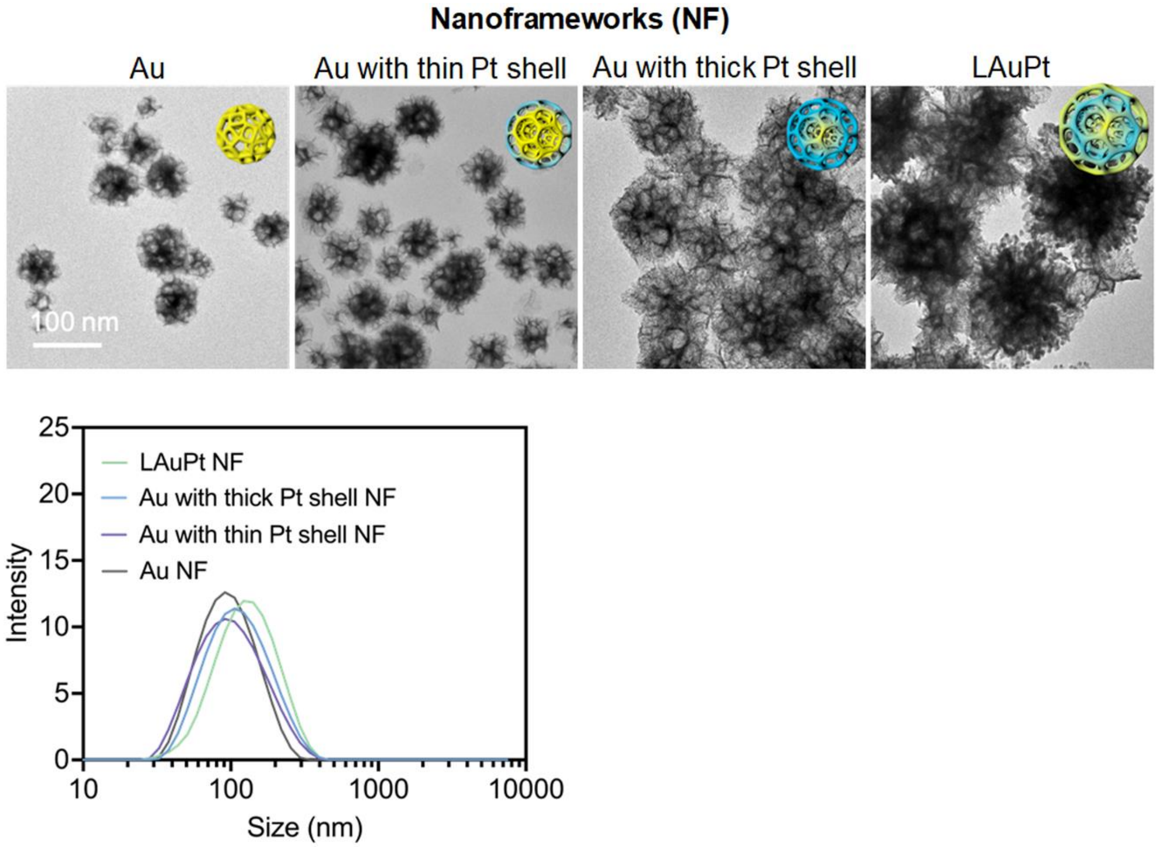

Figure S4: TEM images of the Au, Au with thin Pt shell, Au with thick Pt shell, and LAuPt nanoframeworks (NFs), prepared from different durations of Pt growth (0, 30 min, 2h, 12h) with Chloroplatinic acid.

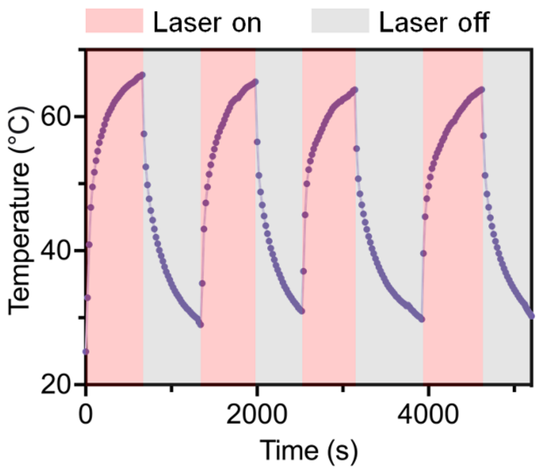

Figure S5: The temperature change of the solution of LAuPt nanoframework (200 μg/mL) over four cycles of NIR-II laser on and off.

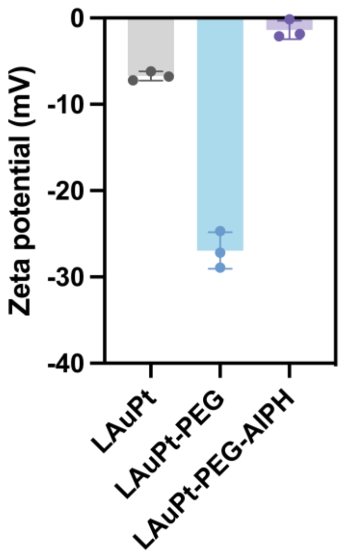

Figure S6: The surface charges of the LAuPt, LAuPt-PEG, and LAuPt-PEG-AIPH nanocomposites measured as the zeta potential. Bar plots: mean ± standard deviation, with data points shown.

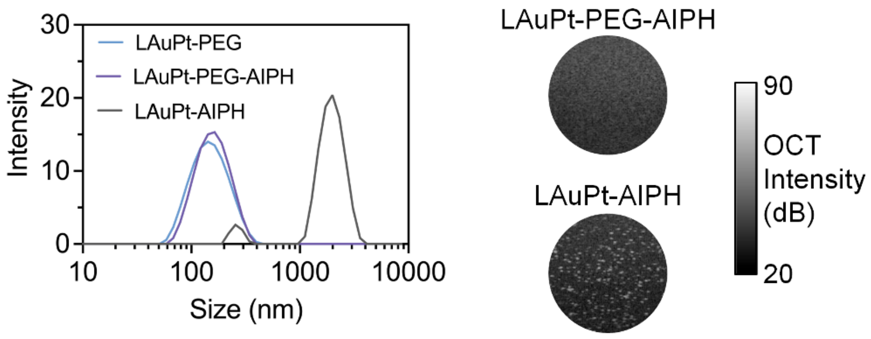

Figure S7: (Left) Size distribution of the LAuPt-PEG, LAuPt-PEG-AIPH, and LAuPt-AIPH. (Right) OCT images of the LAuPt-PEG-AIPH and LAuPt-AIPH show the aggregates from the latter.

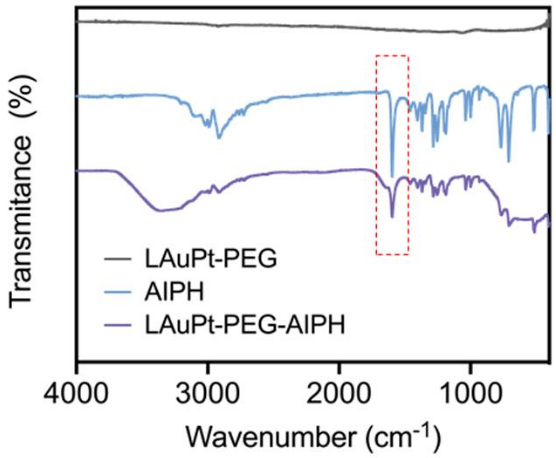

Figure S8: FTIR spectra of the LAuPt-PEG, AIPH, and LAuPt-PEG-AIPH show the loading of AIPH by LAuPt-PEG.

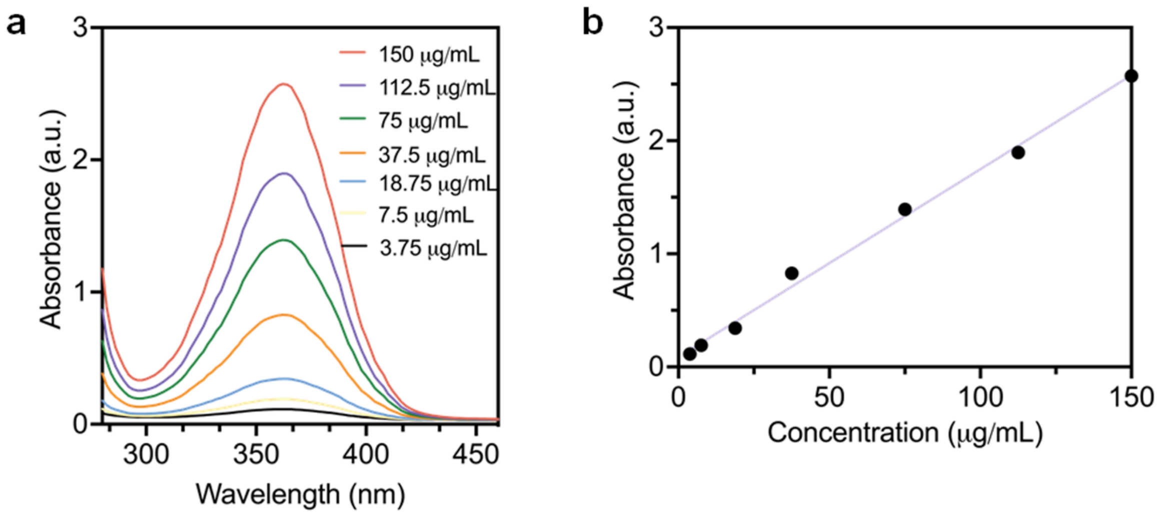

Figure S9: a. Light absorption spectra of AIPH at different concentrations. **b.** The linear relationship between peak absorption of AIPH and its concentration, which was used to estimate the loading capacity of AIPH in LAuPt-PEG.

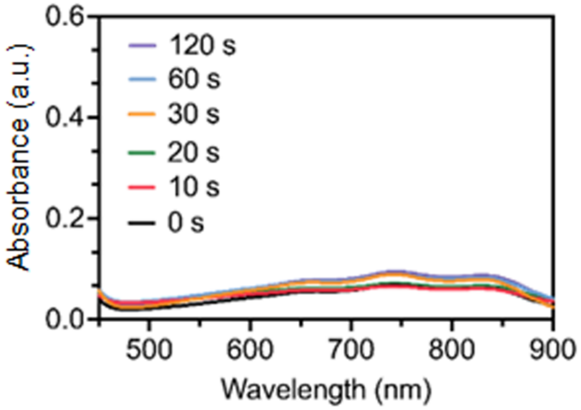

Figure S10: Light absorption spectra of ABTS solution (no LAuPt-PEG-AIPH) with ~1100-1500 nm illumination for different time durations.

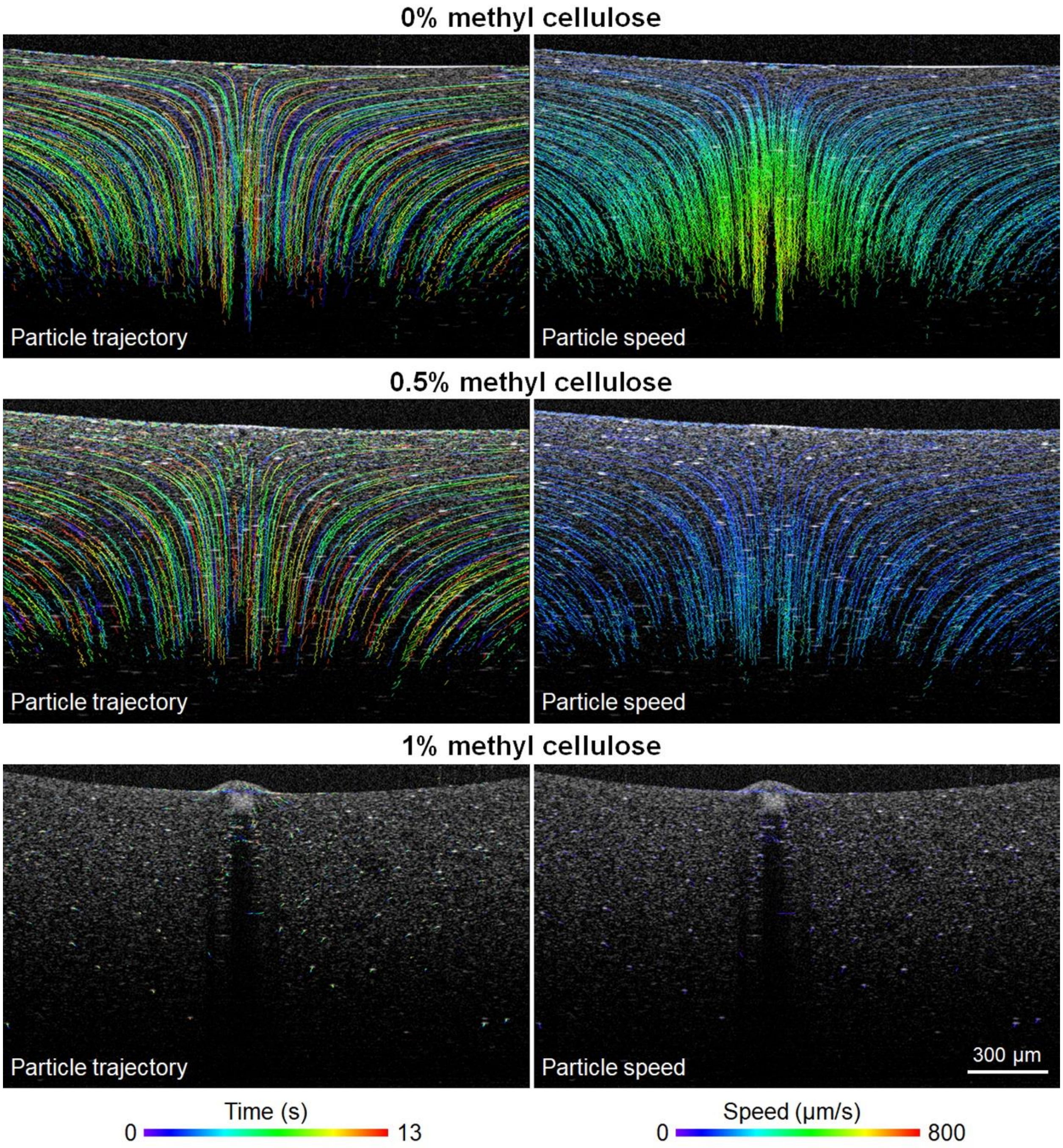

Figure S11: Depth-resolved OCT imaging and measurement of the photothermal dynamics inside the ink with the polystyrene beads concentration as 10% of the one shown in **Figure 4a**. The observation of the process remains the same, suggesting that seeding the ink with the polystyrene beads for the imaging purpose does not affect the conclusion.

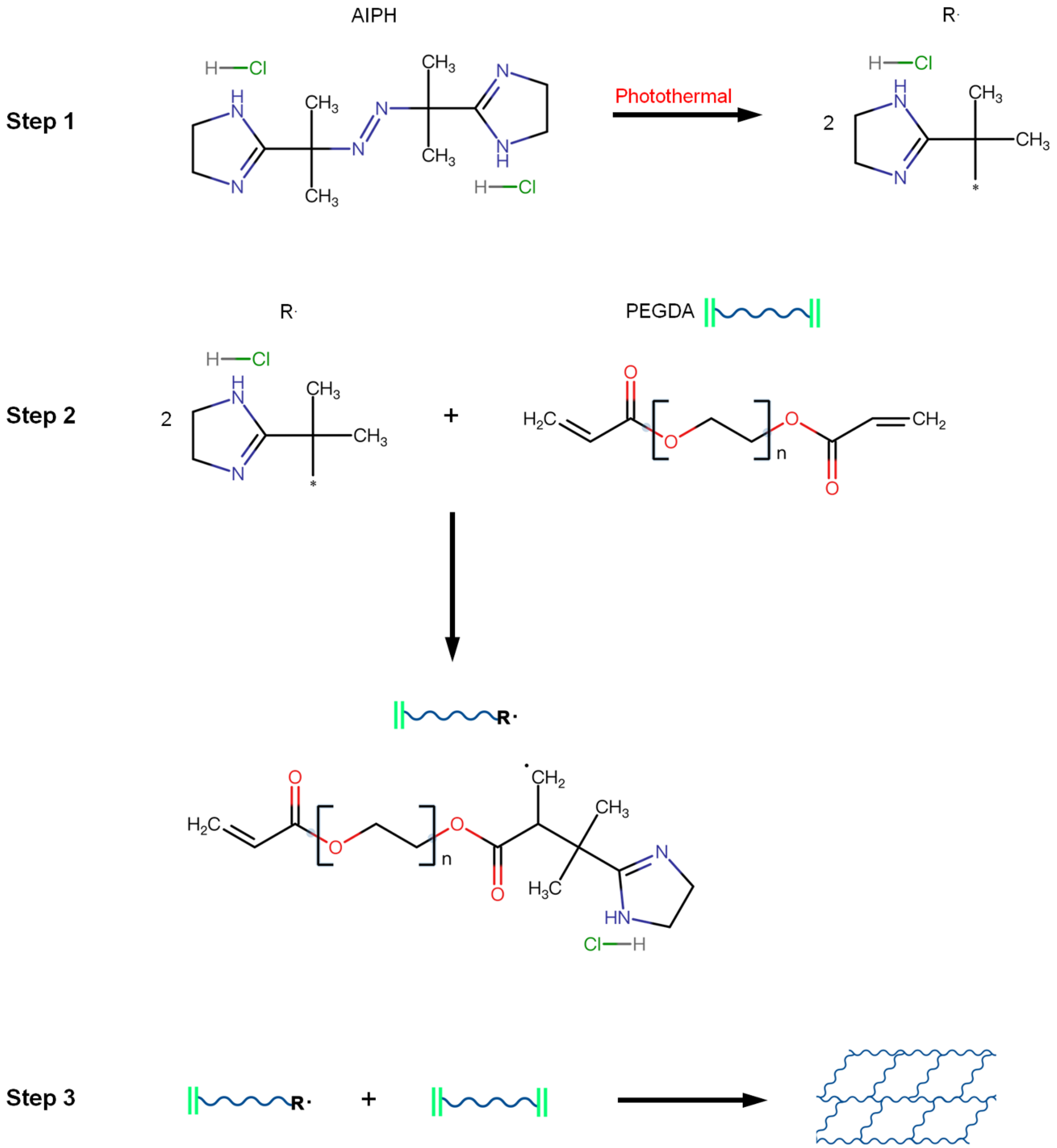

Figure S12: Illustration of the chemical reaction process in the gelation of the ImPSB ink.

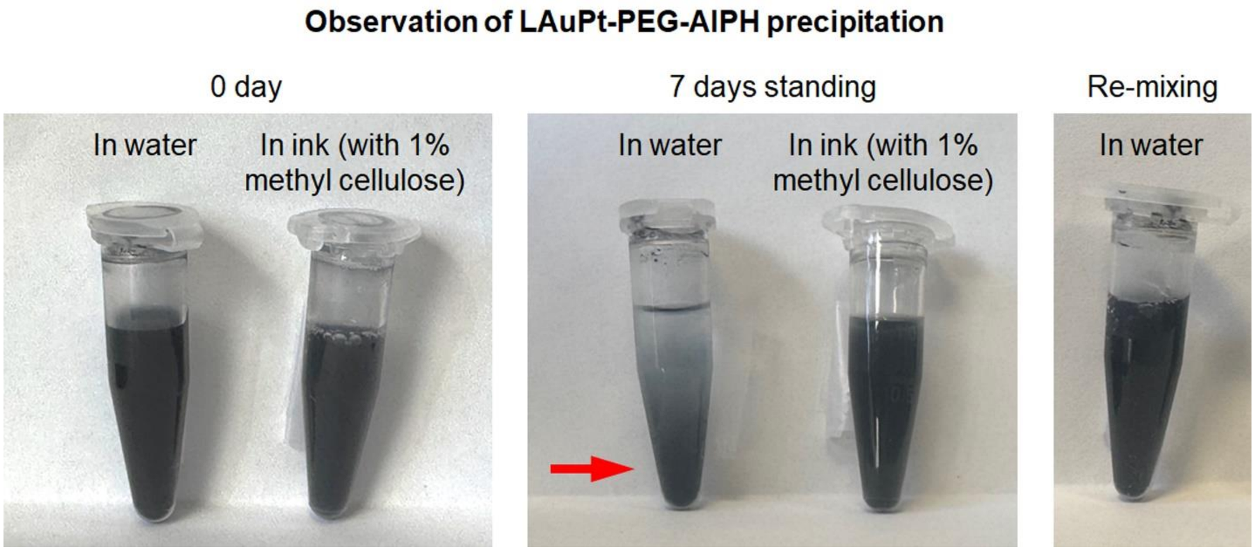

Figure S13: Assessment of precipitation of LAuPt-PEG-AIPH nanocomposite in water and the ink that contains 1% methyl cellulose. After seven days, precipitation appears in water (red arrow) but not in the ink, suggesting 1% methyl cellulose contributes to improving the stability of LAuPt-PEG-AIPH nanocomposite in the aqueous solution. Remixing after precipitation in water well disperses the LAuPt-PEG-AIPH nanocomposite, due to the PEG functionalization of the LAuPt.

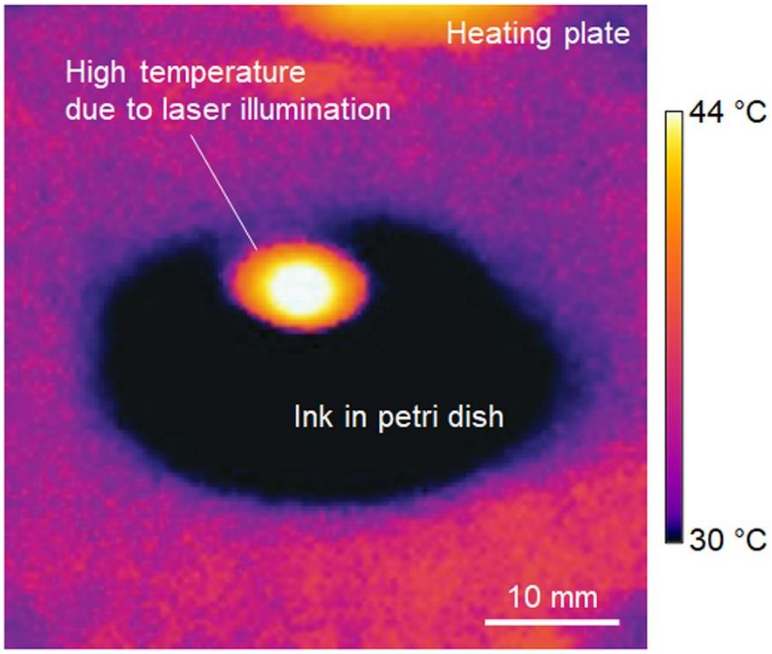

Figure S14: Thermal image of the ink during photothermal gelation shows the temperature increase due to the laser illumination.

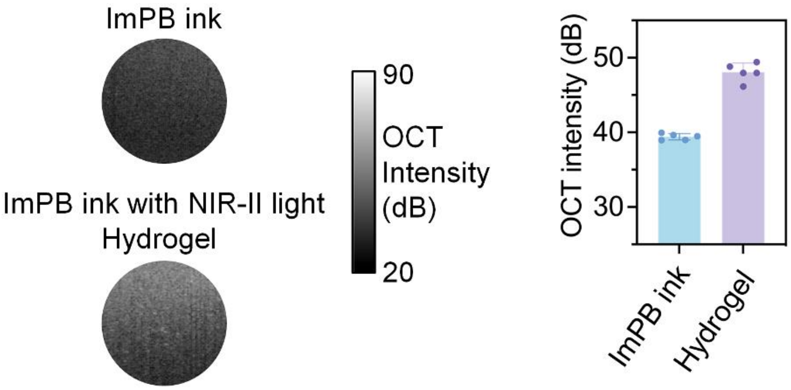

Figure S15: (Left) OCT images of the ImPSB ink and the corresponding hydrogel after ~1100-1500 nm illumination, and (Right) the quantification of the OCT intensity, together show the OCT imaging contrast. Bar plots: mean ± standard deviation, with data points shown.

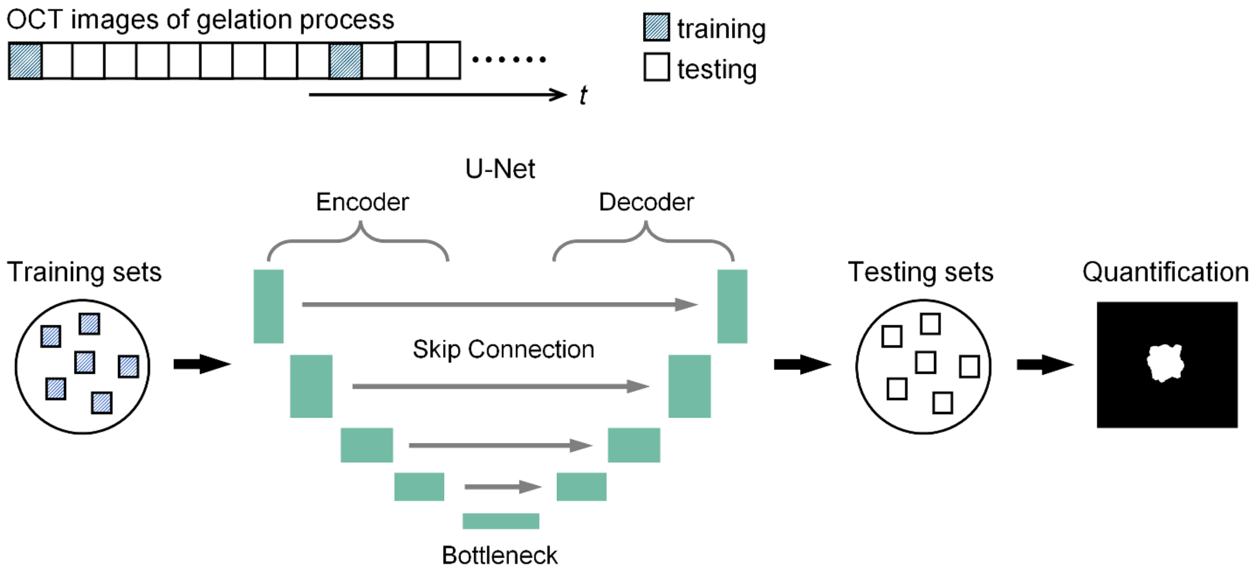

Figure S16: Illustration of the method of training a deep learning neural network (U-Net) for quantification of the ImPSB gelation based on OCT images.

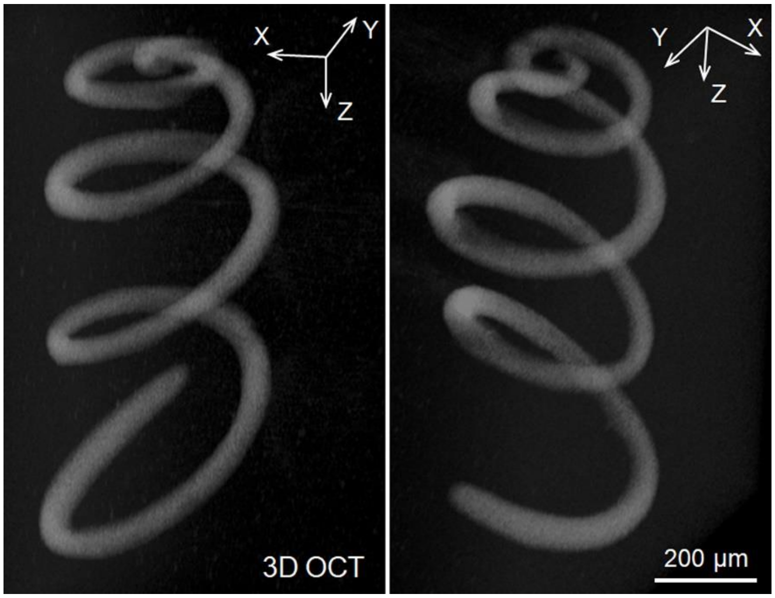

Figure S17: OCT 3D images of the high-resolution 3D spiral structure generated by ImPSB through 4D printing, where a printed spiral line deformed in the z-axis after being placed in the 1X PBS solution. The two views are from different angles.

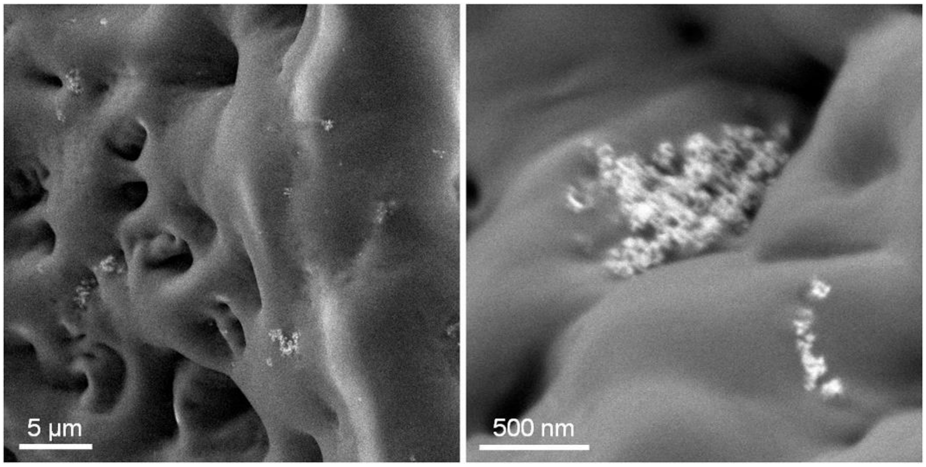

Figure S18: SEM images of printed hydrogel show the hydrogel contains porous LAuPt nanoframeworks remaining with the size of ~100 nm.

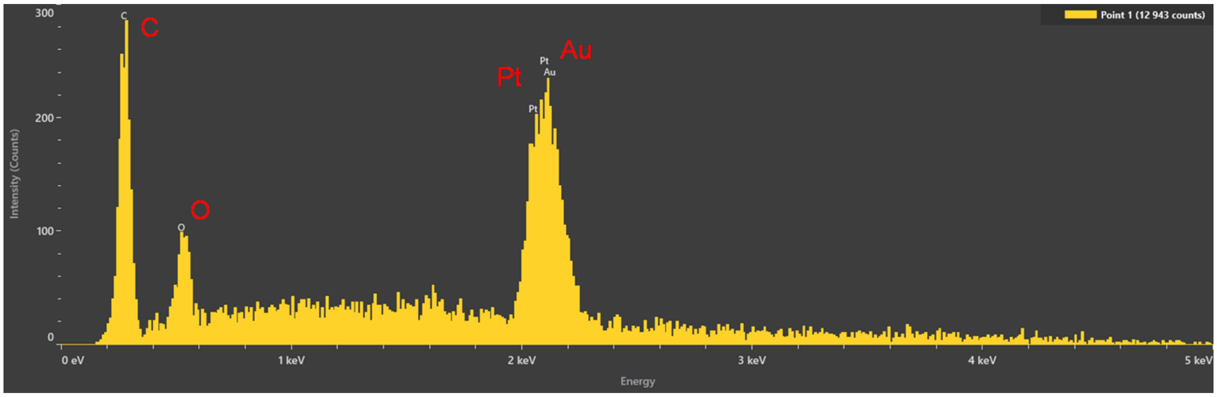

Figure S19: X-ray spectroscopy of printed hydrogel identifies Au and Pt.

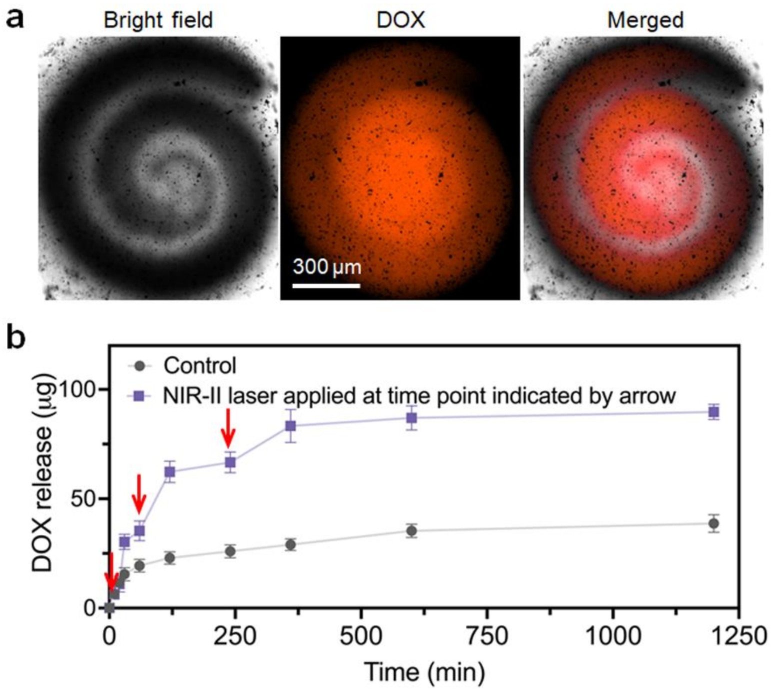

Figure S20: a. Bright-field and fluorescence images of the hydrogel printed with the ink containing doxorubicin (DOX) show the hydrogel can hold DOX. **b.** The photothermal property of the printed hydrogel, enabled by the LAuPt nanoframework in the hydrogel, can be used for a controlled release of DOX through light illumination. A controlled DOX release experiment shows that, in comparison to the natural release of DOX from the printed hydrogel, illumination with ~1100-1500 nm light (red arrows) temporally increases the DOX release rate. Data plots: mean ± standard deviation, printed hydrogel samples N=3.

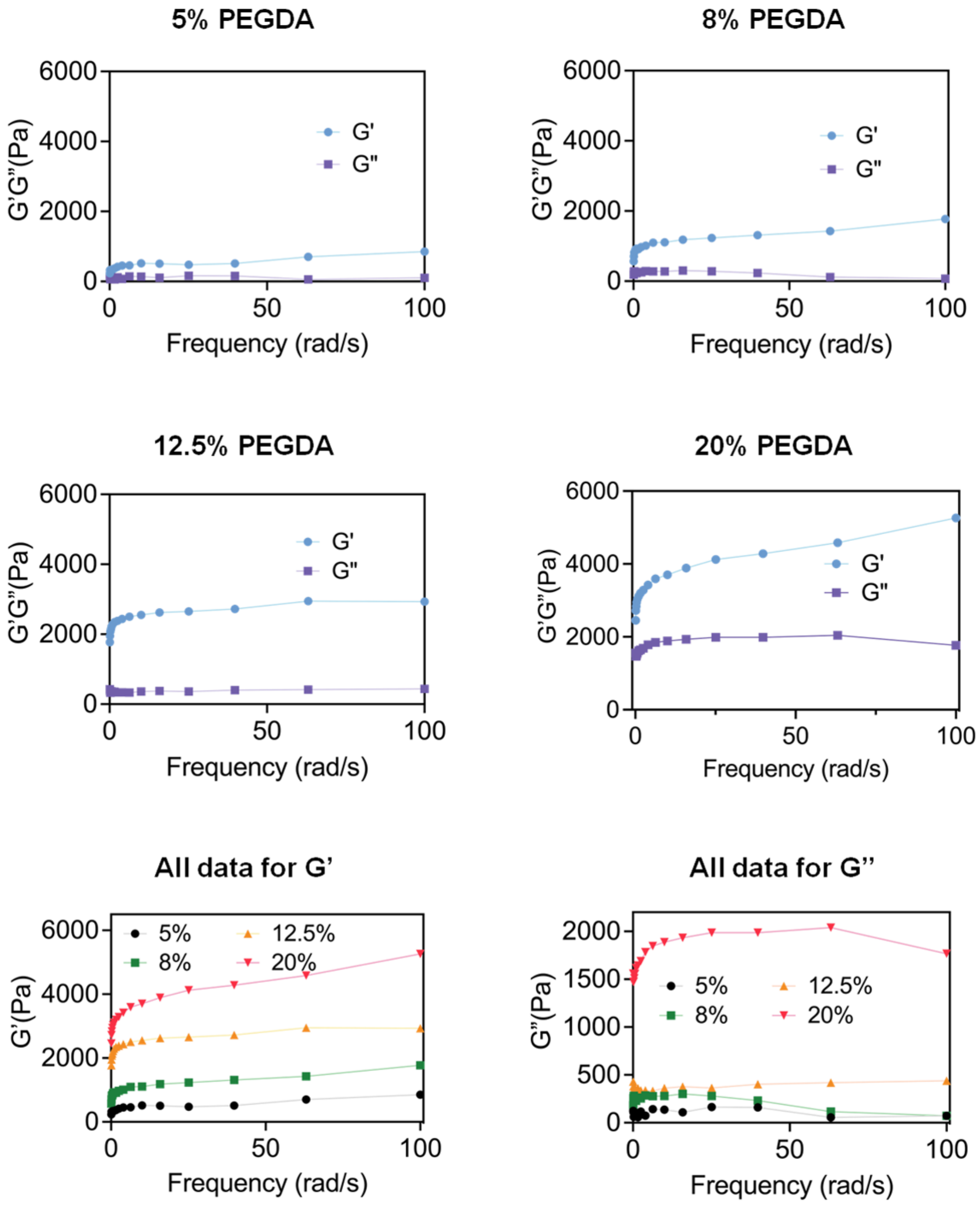

Figure S21: The storage modulus, G', and loss modulus, G", measured by small-amplitude oscillatory shear represent gel-like behavior as evidenced from G'>>G" and both moduli exhibiting little sensitivity to frequency. The gel strength increases with the PEGDA concentration, suggesting that the mechanical properties of the printed hydrogels are tunable based on the PEGDA concentration, covering a large physiological range. The last row are plots for easy comparison between different concentrations of PEGDA.

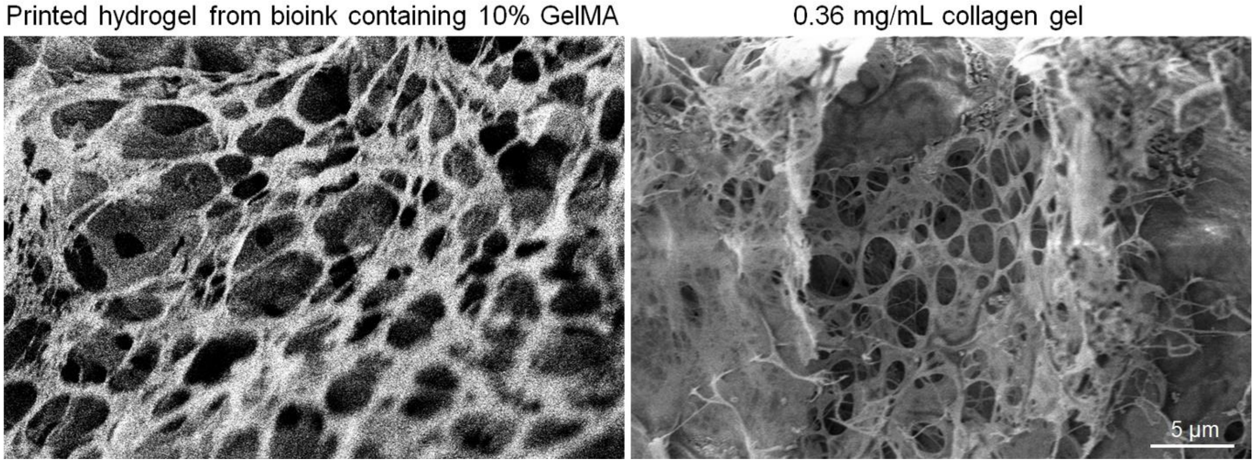

Figure S22: SEM images of the printed hydrogel from bioink containing 10% GelMA and the collagen gel with a concentration of 0.36 mg/mL show similar porous structures.

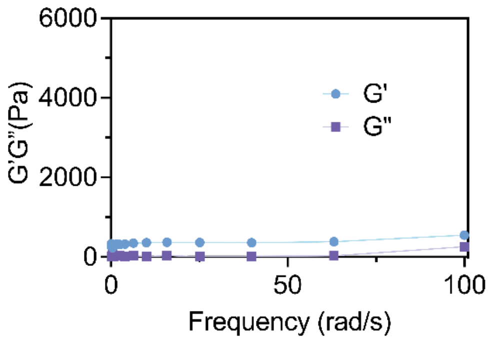

Figure S23: The storage modulus and loss modulus of the hydrogel printed from the ImPSB ink used for cell biocompatibility study, where the PEGDA concentration is 4%, and 10% GelMA is added.

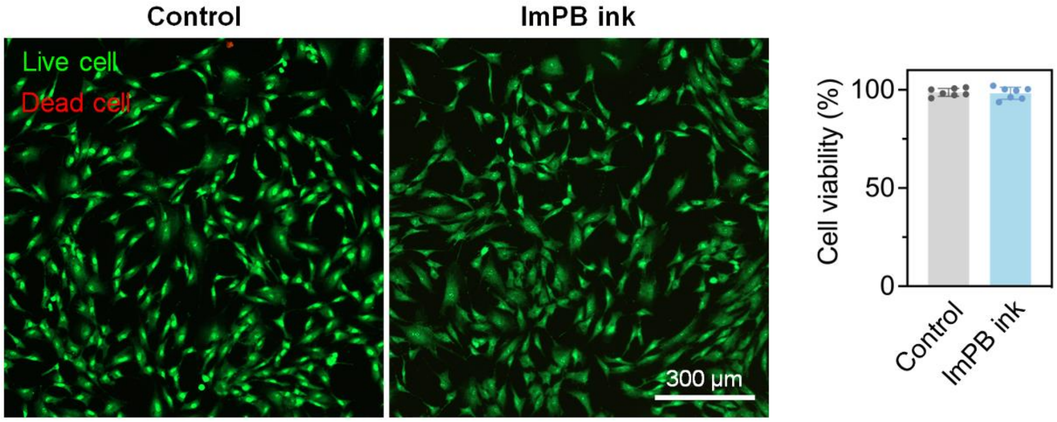

Figure S24: (Left) Live/dead staining and (right) cell viability measurement show the ImPSB ink does not cause cell death. Bar plots: mean ± standard deviation, with data points shown.

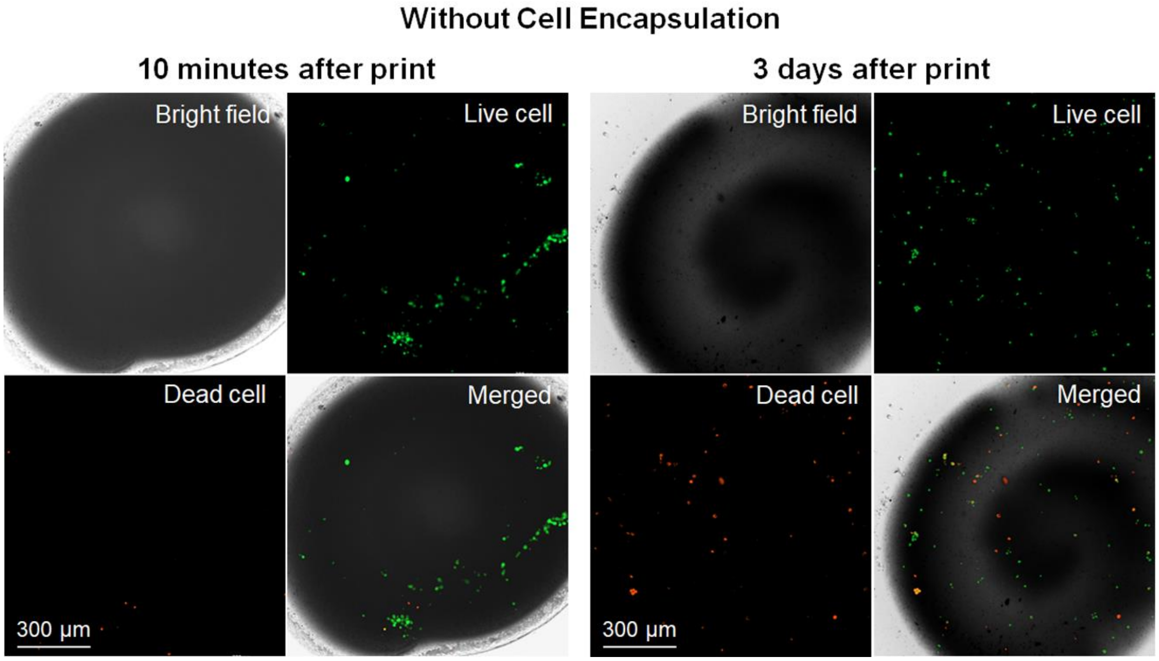

Figure S25: Bright-field images and live/dead cell staining of hydrogels printed with cell-laden bioink without encapsulating the cells (NIH/3T3 cells). The left shows the cell viability 10 minutes after print, and the right shows the cell viability 3 days after print. The reduction of cell viability over time suggests the free alkyl radicals generated from the printing process cause cell death. This justifies the cell encapsulation employed for cell-laden bioprinting in this work.

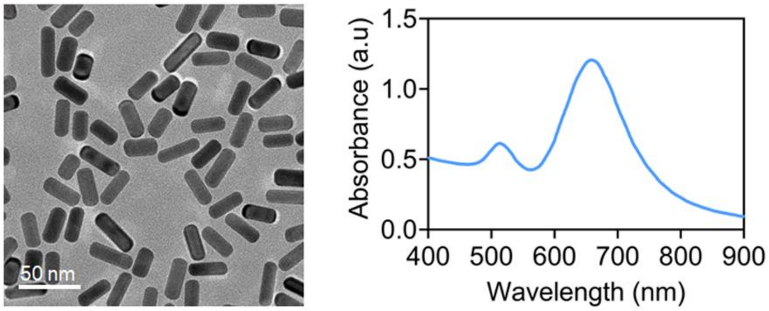

Figure S26: (Left) TEM image of the nanorods. (Right) Absorption spectrum of the nanorods shows the peak absorption at ~660 nm.

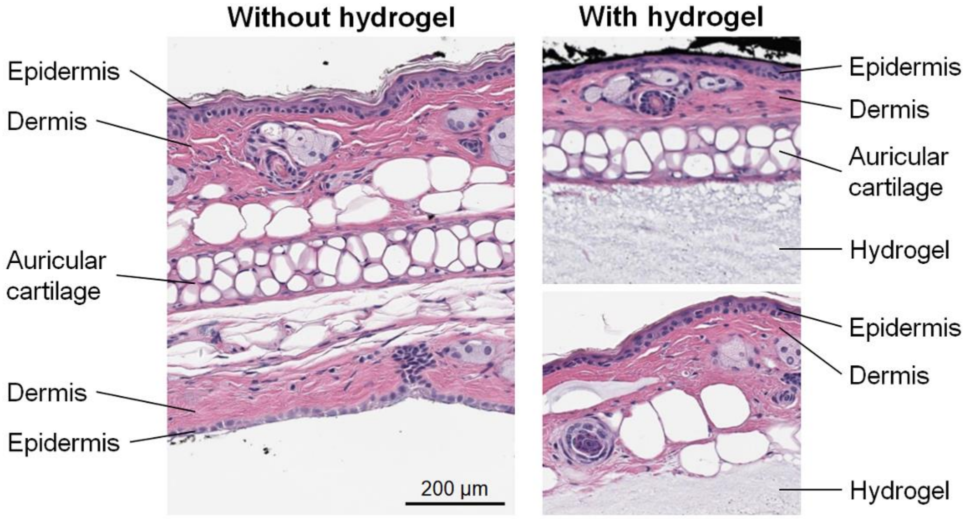

Figure S27: Histology with H&E staining for the mouse ear skin tissues with and without hydrogel. In comparison, the skin tissue with hydrogel has intact structures without noticeable defects, especially at the region interfacing the hydrogel.

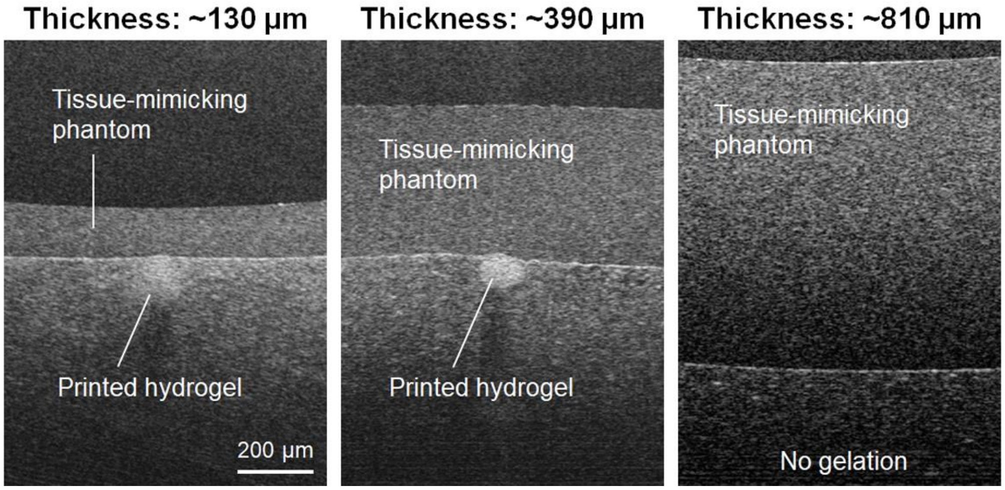

Figure S28: Assessment of the penetration depth of ~40 mW NIR-II printing light for printing through optically scattering tissue-mimicking phantoms of a range of thicknesses. Smooth and continuous line hydrogel can be reliably printed through a thickness of ~130 µm. The printed line of hydrogel can have breaking points through a thickness of ~390 µm. No gelation can be formed through a thickness of ~810 µm.

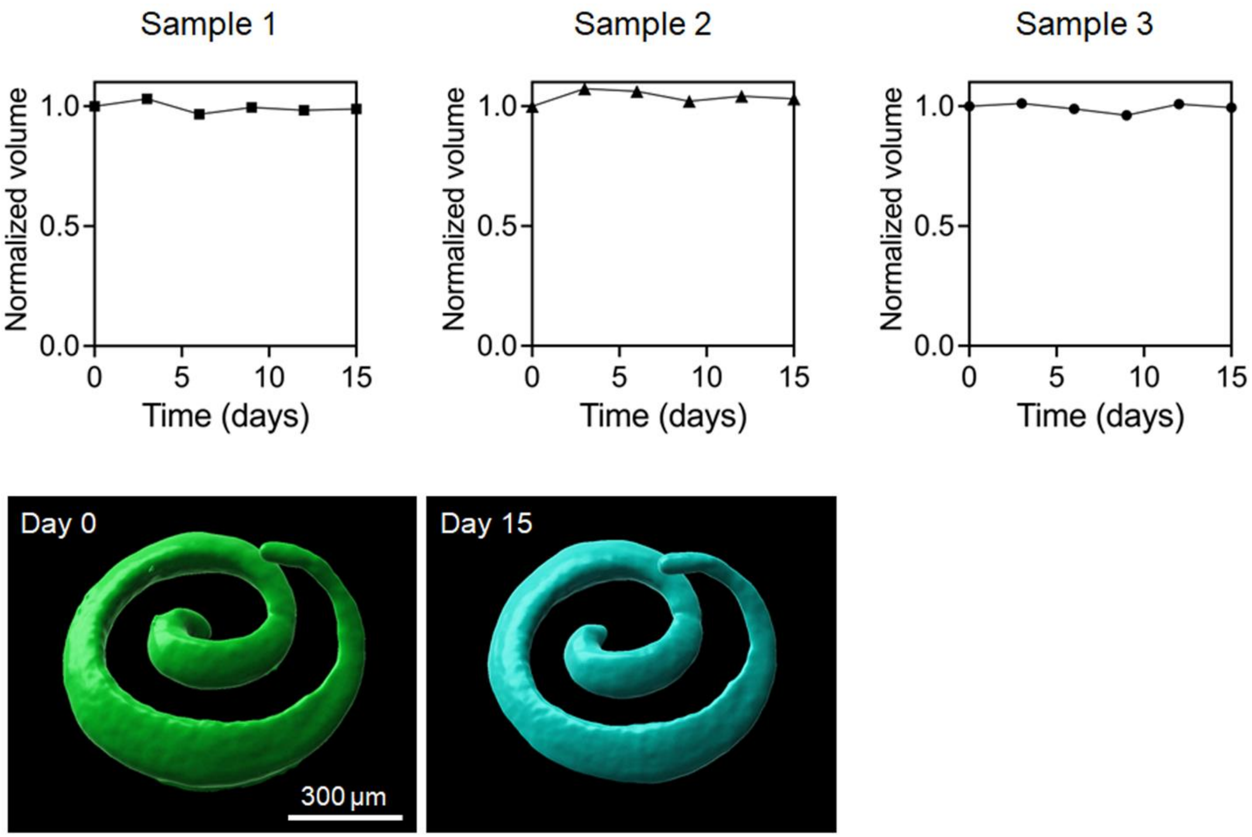

Figure S29: A study of the degradation of the printed hydrogel shows the volumes of three printed hydrogel samples over 15 days in cell culture media, as measured by OCT 3D imaging and the isosurface function in the Imaris software. For each sample, the volume values are normalized to the one at day 0. No degradation of the printed hydrogel is observed over 15 days. The bottom shows two examples of the isosurfaces created from OCT 3D images for a sample at day 0 and day 15.

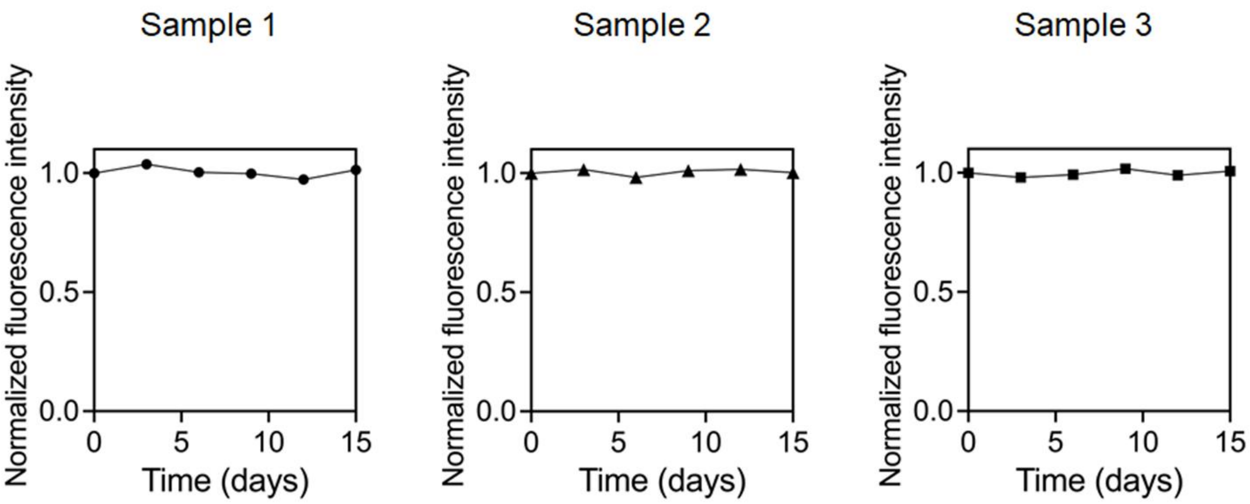

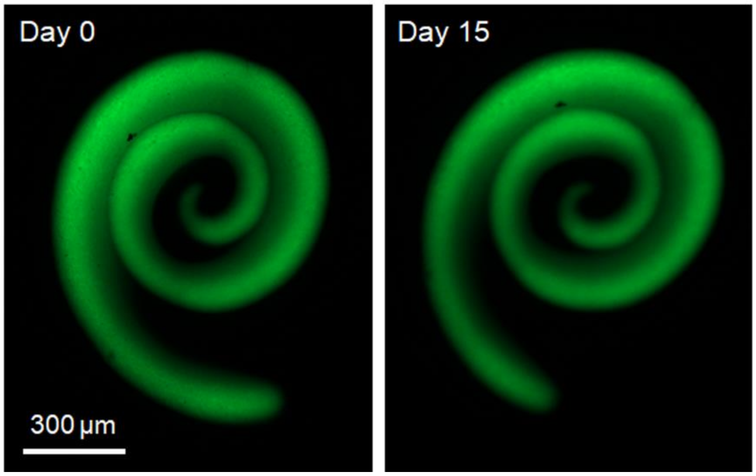

Figure S30: A study of the retention of the LAuPt nanoframeworks within the printed hydrogel shows the intensity of the fluorescence labeling the LAuPt from the entire printed hydrogel over 15 days in cell culture media. For each sample, the fluorescence intensity is normalized to the value at day 0. The LAuPt retention at ~100% is observed over 15 days. The bottom shows two examples of the fluorescence image from the entire hydrogel from a sample at day 0 and day 15.

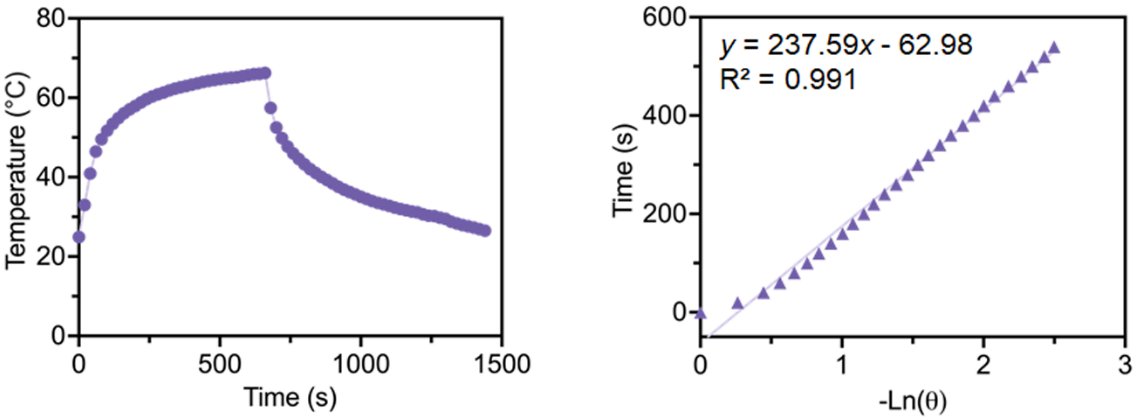

Figure S31: (Left) Temperature over time of aqueous LAuPt solution (200 μg/mL) with ~1100-1500 nm light illumination for 600 seconds. (Right) Plot and linear fit of time versus $-\ln(\theta)$, where $\theta(t) = [T(t) - T_{surroundings}]/\Delta T_{max}$. Here, $T(t)$ is the temperature of solution, $T_{surroundings}$ is the temperature of the environment, and ΔT_{max} is the equilibrium temperature of the solution. The slope of the linear fit is used for the calculation of the photothermal conversion efficiency of LAuPt.

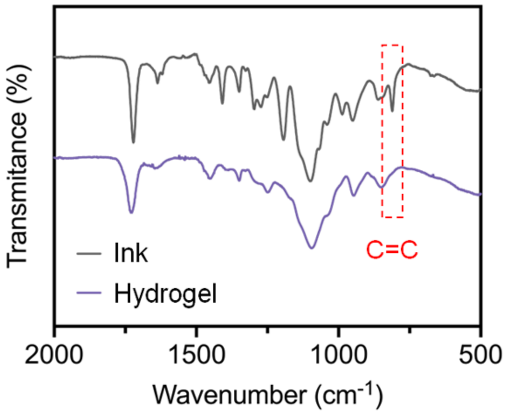

Figure S32: FTIR spectra of the ink and printed hydrogel show the polymerization, which is used for measuring the PEGDA cross-linking efficiency.

Table S1: Implementation of U-Net-based methods.

Methods	Epochs	Loss	Batch Size	LR	Optimizer
U-Net[1]	50	Dice	1	1e-5	RMSprop
UNet++[2]	50	BCE + Dice	1	1e-5	Adam
nnU-Net[3]	50	BCE + Dice	2	1e-4	Adam
Attn U-Net[4]	50	BCE + Dice	1	1e-4	Adam

[1] Ronneberger, Olaf, Philipp Fischer, and Thomas Brox. "U-net: Convolutional networks for biomedical image segmentation." Medical image computing and computer-assisted intervention–MICCAI 2015: 18th international conference, Munich, Germany, October 5-9, 2015, proceedings, part III 18. Springer International Publishing, 2015.

[2] Zhou, Zongwei, et al. "Unet++: A nested u-net architecture for medical image segmentation." Deep Learning in Medical Image Analysis and Multimodal Learning for Clinical Decision Support: 4th International Workshop, DLMIA 2018, and 8th International Workshop, ML-CDS 2018, Held in Conjunction with MICCAI 2018, Granada, Spain, September 20, 2018, Proceedings 4. Springer International Publishing, 2018.

[3] Isensee, F., Jaeger, P. F., Kohl, S. A., Petersen, J., & Maier-Hein, K. H. (2021). nnU-Net: a self-configuring method for deep learning-based biomedical image segmentation. Nature methods, 18(2), 203-211.

[4] Oktay, Ozan, et al. "Attention u-net: Learning where to look for the pancreas." arXiv preprint arXiv:1804.03999 (2018).

Table S2: Results of segmentation. Data are presented with mean ± std from 86 images in testing set. DSC: Dice coefficient; IoU: Intersection over Union; Acc: Accuracy; Spe: specificity; Sens: sensitivity; Pre: precision.

Methods	DSC (%)	IoU (%)	Acc (%)	Spe (%)	Sens (%)	Pre (%)
U-Net	91.04±3.39	83.71±5.35	99.98±0.01	99.99±0.01	91.96±5.29	90.39±4.21
UNet++	89.30±3.88	80.88±6.05	99.97±0.01	99.98±0.01	89.37±8.48	90.00±4.33
nnU-Net	**91.78±2.60**	**84.90±4.32**	**99.98±0.01**	**99.99±0.01**	92.13±5.04	**91.74±4.00**
Attn U-Net	89.88±2.77	81.73±4.41	99.97±0.01	99.98±0.01	**95.49±3.57**	85.13±4.86

Movie S1: A 3D view of LAuPt nanoframework from TEM tomography shows its porous structure. This movie is associated with **Figure 3**.

Movie S2: Time-lapse OCT imaging of the fluid dynamics in the ink containing 0% methyl cellulose upon point illumination with ~1100-1500 nm light shows high-speed fluid flows. This movie is associated with **Figure 4**.

Movie S3: Time-lapse OCT imaging of the fluid dynamics in the ink containing 0.5% methyl cellulose upon point illumination with ~1100-1500 nm light shows reduced fluid flows. This movie is associated with **Figure 4**.

Movie S4: Time-lapse OCT imaging of the fluid dynamics in the ink containing 1% methyl cellulose upon point illumination with ~1100-1500 nm light shows absence of fluid flows and localized gelation process. This movie is associated with **Figure 4**.

Movie S5: Time-lapse 3D OCT imaging of the printing process of a spiral-shaped hydrogel. This movie is associated with **Figure 5**.
